# Supplementary material for: Polygenic prediction of coronary heart disease among 130,000 Mexican adults
Source: Eur J Prev Cardiol. Author manuscript; Available in PMC 2026 Feb 18. (PMC7618473; doi:10.1093/eurjpc/zwaf728)
Supplement: Graphical Abstract [file EMS211407-supplement-Graphical_Abstract.pdf]

Construct polygenic risk scores (PRS) for CHD from a large population-based cohort of Mexican adults

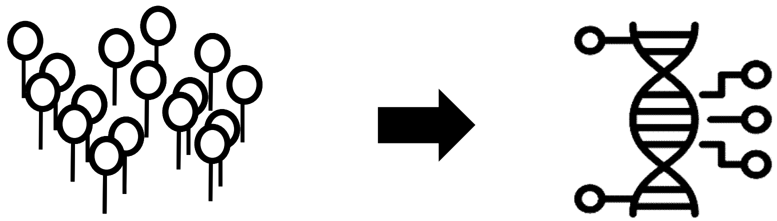

Estimate associations between each PRS and CHD risk

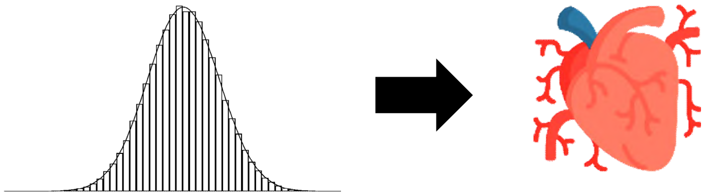

Compare European versus Multi-ancestry scores in men versus women

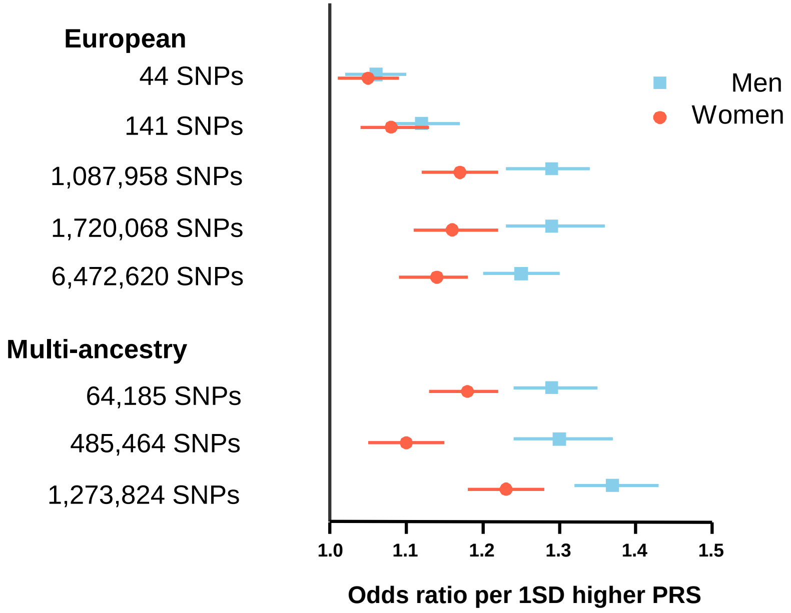

CHD=coronary heart disease, PRS=polygenic risk score SNP=single nucleotide polymorphism
