# Supplementary material for: Polygenic prediction of coronary heart disease among 130,000 Mexican adults
Source: Eur J Prev Cardiol. Author manuscript; Available in PMC 2026 Feb 18. (PMC7618473; doi:10.1093/eurjpc/zwaf728)
Supplement: Online Appendix [file EMS211407-supplement-Online_Appendix.pdf]

Polygenic prediction of coronary heart disease among 130,000 Mexican adults

Online appendix

Page

Webtables

1. CHD polygenic risk scores selected for evaluation 2
2. Baseline characteristics of 133,207 participants aged 35-79 years, by district 3
3. Baseline characteristics of 137,391 participants aged 35-89 years 4

Baseline characteristics of 133,207 participants aged 35-79 years by fifth of PRS by:

4. Tada *et al.* 5
5. Oni-Orisan *et al.* 6
6. Koyama *et al.* 7
7. Tcheandjieu *et al.* 8
8. Tamlander *et al.* 9
9. Patel *et al.* 10
10. Inouye *et al.* 11
11. Khera *et al.* 12

Webfigures

1. Pairwise correlations between the eight selected PRS 13

Odds of CHD per 1SD increase in each PRS:

2. By baseline age 14
3. By highest level of education 15
4. By waist-to-hip ratio 16
5. By systolic blood pressure 17
6. By diastolic blood pressure 18
7. By smoking status 19
8. By baseline diabetes status 20
9. By level of Indigenous American ancestry 21

Odds ratios per 1SD increase in each PRS among participants aged 35-79 years at recruitment, for the outcome of:

10. Baseline self-reported angina 22
11. Baseline self-reported heart attack 23
12. Baseline self-reported angina *or* heart attack 24
13. Baseline self-reported angina or heart attack, *or* death before age 80 with CHD listed as the *primary* cause of death 25
14. Death before age 80 with CHD listed *anywhere* on the death certificate 26
15. Death before age 80 with CHD listed as the *primary* cause on the death certificate 27

Sensitivity analyses

16. Varying the CHD outcome, with partial adjustments 28
17. Varying the CHD outcome, with full adjustments 29
18. Odds of CHD (varying CHD outcomes) per 1SD increase for the Patel *et al.* PRS, by sex 30
19. Odds of CHD (primary definition) among participants unrelated to the 3rd degree 31
20. Odds of CHD before age 90 years per 1SD increase in each PRS, among participants aged 35-89 years at recruitment 32
21. Odds of CHD before age 90 years by fifth of each PRS, among participants aged 35-89 years at recruitment 33

Webtable 1: CHD polygenic risk scores selected for evaluation

Authors / PRS ID in PGS catalogue/Ancestry	PRS characteristics	CHD odds or hazard ratio (95% CI)*	AUC (95%CI)
Tada et al[w1] /PGS000011/European	A PRS constructed using 50 SNPs and European GWAS. **GWAS:** European dominant (~95%) with a small proportion of Asian samples **Evaluation sample:** 100% European	HR per SD: 1.23 (1.18-1.28)	0.75 (no CI reported)
Oni-orisan et al[w2] /PGS004595/European	A PRS constructed using European GWAS and 164 genome-wide significant CHD risk SNPs from UKB and CC4D GWAS. It was evaluated among datasets that contains Hispanic participants (n=1538). **GWAS:** European dominant **Evaluation sample:** Multi-ancestry	HR per SD: 1.87 (1.19-2.95)	NR
Koyama et al[w3] /PGS000337/Japanese and European	A trans-ancestry PRS that used Japanese and European genetic information during construction. **GWAS:** European and Japanese dominant **Training sample:** Japanese dominant **Evaluation sample:** Japanese dominant	OR per SD: 1.84 (1.74-1.94)	0.67 (0.66–0.69)
Tcheandjieu et al[w4] /PGS003446/Multi-ancestry	A trans-ancestry PRS that used Hispanic, European and Japanese genetic information during construction using pruning and thresholding [w5]. **GWAS:** Multi-ancestry (Hispanics, African, Japanese, European) **Training sample:** European, African and Hispanics **Evaluation sample:** Hispanic	OR per SD: 1.43 (1.27-1.61)	NR
Tamlander et al[w6] / PGS001780/European	A PRS developed using a novel method, PRS-CS-auto. **GWAS:** European dominant (~80%) with a small proportion of Asian samples **Evaluation sample:** European	OR per SD (in UK Biobank): 1.72 (1.70-1.75)	0.79 (0.79–0.80)
Patel et al[w7] /PGS003725/Multi-ancestry	A trans-ancestry and multi-trait PRS derived using Hispanic, European, African and Asian genetic information. **GWAS:** Multi-ancestry (Hispanics, African, Japanese, European) **Training sample:** European **Evaluation sample:** Hispanic	OR per SD: 1.61 (1.53-1.70)	NR
Inouye et al[w8] /PGS000018/European	A meta-score based on the weighted average of 3 PRSs. **GWAS:** European dominant (~90%) with a small proportion of Asian samples **Training sample:** European dominant **Evaluation sample**: European dominant	HR per SD: 1.71 (1.68-1.73)	0.79 (no CI reported)
Khera et al[w9] /PGS000013/European	A PRS developed using LDPred2[w10] with a large European dominant GWAS input. **GWAS:** European dominant (~80%) with a small proportion of Asian samples **Training sample:** European dominant **Evaluation sample:** European dominant	OR per SD (in training sample): 1.72 (1.67-1.78)	0.81 (0.81-0.81)

* In the 'evaluation sample' unless otherwise stated. AUC=Area under the receiver operating characteristic curve. CHD=Coronary heart disease. CI=Confidence interval. OR= Odds ratio. HR=Hazard ratio. NR=Not reported

Reference

w1. Tada H, Melander O, Louie JZ, Catanese JJ, Rowland CM, Devlin JJ, et al. Risk prediction by genetic risk scores for coronary heart disease is independent of self-reported family history. *Eur Heart J*. 2016;37(6):561-567.

w2. Oni-Orisan A, Haldar T, Cayabyab MAS, Ranatunga DK, Hoffmann TJ, Iribarren C, et al. Polygenic Risk Score and Statin Relative Risk Reduction for Primary Prevention of Myocardial Infarction in a Real-World Population. *Clin Pharmacol Ther*. 2022;112(5):1070-1078.

w3. Koyama S, Ito K, Terao C, Akiyama M, Horikoshi M, Momozawa Y, et al. Population-specific and trans-ancestry genome-wide analyses identify distinct and shared genetic risk loci for coronary artery disease. *Nature genetics*. 2020;52(11):1169-1177.

w4. Tcheandjieu C, Zhu X, Hilliard AT, Clarke SL, Napolioni V, Ma S, et al. Large-scale genome-wide association study of coronary artery disease in genetically diverse populations. *Nat Med*. 2022;28(8):1679-1692.

w5. Choi SW, O'Reilly PF. PRSice-2: Polygenic Risk Score software for biobank-scale data. *GigaScience*. 2019;8(7).

w6. Tamlander M, Mars N, Pirinen M, FinnGen, Widen E, Ripatti S. Integration of questionnaire-based risk factors improves polygenic risk scores for human coronary heart disease and type 2 diabetes. *Communications Biology*. 2022;5(1):158.

w7. Patel AP, Wang M, Ruan Y, Koyama S, Clarke SL, Yang X, et al. A multi-ancestry polygenic risk score improves risk prediction for coronary artery disease. *Nature Medicine*. 2023;29(7):1793-1803.

w8. Inouye M, Abraham G, Nelson CP, Wood AM, Sweeting MJ, Dudbridge F, et al. Genomic Risk Prediction of Coronary Artery Disease in 480,000 Adults: Implications for Primary Prevention. *J Am Coll Cardiol*. 2018;72(16):1883-1893.

w9. Khera AV, Chaffin M, Aragam KG, Haas ME, Roselli C, Choi SH, et al. Genome-wide polygenic scores for common diseases identify individuals with risk equivalent to monogenic mutations. *Nature genetics*. 2018;50(9):1219-1224.

w10. Privé F, Arbel J, Vilhjálmsson BJ. LDpred2: better, faster, stronger. *Bioinformatics*. 2021;36(22-23):5424-5431.

Webtable 2: Baseline characteristics of 133,207 participants, by study district

	Iztapalapa *n*=82,192 (62%)	Coyoacán *n*=51,015 (38%)	All n=133,207
**Men**	25,380 (31%)	17,958 (35%)	43,338 (33%)
**Age, years**	51.3 (12.0)	51.2 (11.5)	51.2 (11.8)
**Unrelated participants**	43,772 (53%)	32,103 (63%)	75,875 (57%)
**Ancestry admixture percentage**			
Indigenous American	68.9 (17.2)	63.5 (18.4)	66.8 (17.9)
African	3.3 (2.8)	3.7 (2.8)	3.4 (2.8)
East Asian	1.4 (1.5)	1.5 (2.3)	1.4 (1.8)
European	26.4 (15.4)	31.4 (16.9)	28.3 (16.2)
**Highest attained educational level**			
University/high school	10,085 (12%)	10,624 (21%)	20,709 (16%)
Middle school	20,585 (25%)	12,847 (25%)	33,432 (25%)
Elementary	40,821 (50%)	22,086 (43%)	62,907 (47%)
Other	10,651 (13%)	5,440 (11%)	16,091 (12%)
Missing	50 (0%)	18 (0%)	68 (0%)
**Smoking status**			
Never	41,904 (51%)	22,687 (45%)	64,591 (48%)
Former	15,545 (19%)	10,631 (21%)	26,176 (20%)
Current	24,698 (30%)	17,632 (35%)	42,330 (32%)
Missing	45 (0%)	65 (0%)	110 (0%)
**Alcohol intake**			
Never	18,222 (22%)	7,881 (15%)	26,103 (20%)
Former	11,688 (14%)	6,563 (13%)	18,251 (14%)
Current	52,261 (64%)	36,542 (72%)	88,803 (67%)
Missing	21 (0%)	29 (0%)	50 (0%)
**Physical measures**			
SBP, mmHg	126.5 (16.4)	128.5 (16.9)	127.2 (16.6)
DBP, mmHg	82.6 (10.0)	84.0 (10.3)	83.1 (10.2)
BMI, kg/m²	29.5 (5.1)	28.5 (4.9)	29.1 (5.1)
Waist-to-hip Ratio	0.91 (0.08)	0.90 (0.08)	0.90 (0.08)
**Laboratory measurements**			
HDL-C, mmol/L	0.99 (0.21)	1.02 (0.22)	1.00 (0.21)
LDL-C, mmol/L	2.45 (0.78)	2.47 (0.80)	2.46 (0.79)
Triglycerides, mmol/L	1.57 (0.67)	1.57 (0.64)	1.57 (0.66)
HbA1c, %	6.22 (1.76)	5.90 (1.61)	6.10 (1.71)
eGFR, ml/min/1.73m²*	101.3 (16.4)	102.2 (15.4)	101.7 (16.0)
**Prior disease †**			
Coronary heart disease	1,008 (1%)	893 (2%)	1,901 (1%)
Stroke	777 (1%)	637 (1%)	1,414 (1%)
Cancer	838 (1%)	742 (1%)	1,580 (1%)
Diabetes ‡	16,663 (20%)	8,133 (16%)	24,796 (19%)
Other §	5,109 (6%)	6,004 (12%)	11,113 (8%)

Numbers are n (%) or mean (SD). SBP=systolic blood pressure, DBP=diastolic blood pressure, BMI=body mass index, HDL-C=high density lipoprotein cholesterol, LDL-C=low density lipoprotein cholesterol, HbA1c=glycosylated haemoglobin A1c

* Calculated using the 2021 CKD-EPI equation, based on NMR-measured creatinine levels.

† Self-reported previous diagnoses unless otherwise stated.

‡ Self-reported previously-diagnosed diabetes or glycosylated haemoglobin ≥6.5%.

§ Other diseases include self-reported emphysema, chronic kidney disease, peptic ulcer, liver cirrhosis, and peripheral arterial disease.

Webtable 3: Baseline characteristics of 137,391 participants aged 35-89 years

	**Men n=44,853 (33%)**	**Women n=92,538 (67%)**	**All n=137,391**
**Age, years**	53.1 (13.1)	51.8 (12.8)	52.2 (12.9)
**Resident of Coyoacán**	18,425 (41%)	33,947 (37%)	52,372 (38%)
**Unrelated participants**	24,979 (56%)	52,896 (57%)	77,875 (57%)
**Ancestry admixture percentage**			
Indigenous American	66.4 (18.1)	67.0 (17.9)	66.8 (17.9)
African	3.4 (2.8)	3.5 (2.8)	3.4 (2.8)
East Asian	1.4 (2.0)	1.4 (1.7)	1.4 (1.8)
European	28.7 (16.4)	28.2 (16.2)	28.4 (16.2)
**Highest attained educational level**			
University/high school	10,443 (23%)	10,421 (11%)	20,864 (15%)
Middle school	11,596 (26%)	22,116 (24%)	33,712 (25%)
Elementary	18,694 (42%)	46,226 (50%)	64,920 (47%)
Other	4,104 (9%)	13,716 (15%)	17,820 (13%)
Missing	16 (0%)	59 (0%)	75 (0%)
**Smoking status**			
Never	9,084 (20%)	57,951 (63%)	67,035 (49%)
Former	13,876 (31%)	13,553 (15%)	27,429 (20%)
Current	21,852 (49%)	20,958 (23%)	42,810 (31%)
Missing	41 (0%)	76 (0%)	117 (0%)
**Alcohol intake**			
Never	2,859 (6%)	24,582 (27%)	27,441 (20%)
Former	8,179 (18%)	11,160 (12%)	19,339 (14%)
Current	33,793 (75%)	56,763 (61%)	90,556 (66%)
Missing	22 (0%)	33 (0%)	55 (0%)
**Physical measures**			
SBP, mmHg	128.8 (15.9)	127.0 (17.2)	127.6 (16.8)
DBP, mmHg	84.4 (10.0)	82.5 (10.3)	83.1 (10.2)
BMI, kg/m²	27.9 (4.3)	29.6 (5.3)	29.0 (5.1)
Waist-to-hip Ratio	0.95 (0.07)	0.88 (0.07)	0.90 (0.08)
**Laboratory measurements**			
HDL-C, mmol/L	0.93 (0.19)	1.04 (0.22)	1.00 (0.21)
LDL-C, mmol/L	2.38 (0.79)	2.49 (0.79)	2.45 (0.79)
Triglycerides, mmol/L	1.64 (0.68)	1.53 (0.64)	1.57 (0.66)
HbA1c, %	6.10 (1.70)	6.10 (1.70)	6.10 (1.70)
eGFR, ml/min/1.73m²*	100.3 (16.4)	101.3 (16.6)	100.9 (16.5)
**Prior disease †**			
Coronary heart disease	937 (2%)	1,152 (1%)	2,089 (2%)
Stroke	547 (1%)	1,011 (1%)	1,558 (1%)
Cancer	311 (1%)	1,377 (1%)	1,688 (1%)
Diabetes ‡	8,599 (19%)	17,242 (19%)	25,841 (19%)
Other §	2,449 (5%)	9,128 (10%)	11,577 (8%)

Numbers are n (%) or mean (SD). SBP=systolic blood pressure, DBP=diastolic blood pressure, BMI=body mass index, HDL-C=high density lipoprotein cholesterol, LDL-C=low density lipoprotein cholesterol, HbA1c=glycosylated haemoglobin A1c

* Calculated using the 2021 CKD-EPI equation, based on NMR-measured creatinine levels.

† Self-reported previous diagnoses unless otherwise stated.

‡ Self-reported previously-diagnosed diabetes or glycosylated haemoglobin ≥6.5%.

§ Other diseases include self-reported emphysema, chronic kidney disease, peptic ulcer, liver cirrhosis, and peripheral arterial disease.

Webtable 4: Baseline characteristics of 133,207 participants aged 35-79 by fifth of PRS Tada *et al.*

Tada et al/PGS000011/ European/44 SNPs	Fifth of the PRS distribution					All n=133,207
	1 n=26,642 (20%)	2 n=26,641 (20%)	3 n=26,641 (20%)	4 n=26,641 (20%)	5 n=26,642 (20%)	
**Age, years**	51.3 (11.9)	51.3 (11.9)	51.2 (11.9)	51.2 (11.8)	51.2 (11.7)	51.2 (11.8)
**Resident of Coyoacán**	10,079 (38%)	10,295 (39%)	10,077 (38%)	10,257 (39%)	10,307 (39%)	51,015 (38%)
**Ancestry admixture percentage**						
Indigenous American	67.1 (17.5)	66.8 (17.9)	66.7 (18.0)	67.0 (18.0)	66.6 (17.8)	66.8 (17.9)
African	3.5 (3.0)	3.5 (2.9)	3.4 (2.8)	3.4 (2.7)	3.4 (2.7)	3.4 (2.8)
East Asian	1.4 (1.8)	1.4 (1.6)	1.4 (2.0)	1.4 (1.8)	1.4 (1.9)	1.4 (1.8)
European	27.9 (15.8)	28.4 (16.3)	28.4 (16.3)	28.2 (16.3)	28.6 (16.1)	28.3 (16.2)
**Highest attained educational level**						
University/high school	4,091 (15%)	4,164 (16%)	4,143 (16%)	4,183 (16%)	4,128 (16%)	20,709 (16%)
Middle school	6,695 (25%)	6,776 (25%)	6,560 (25%)	6,613 (25%)	6,788 (25%)	33,432 (25%)
Elementary	12,557 (47%)	12,444 (47%)	12,679 (48%)	12,668 (48%)	12,559 (47%)	62,907 (47%)
Other	3,282 (12%)	3,244 (12%)	3,244 (12%)	3,167 (12%)	3,154 (12%)	16,091 (12%)
Missing	17 (0%)	13 (0%)	15 (0%)	10 (0%)	13 (0%)	68 (0%)
**Smoking status**						
Never	12,874 (48%)	12,888 (48%)	12,929 (49%)	13,051 (49%)	12,849 (48%)	64,591 (48%)
Former	5,199 (20%)	5,156 (19%)	5,225 (20%)	5,213 (20%)	5,383 (20%)	26,176 (20%)
Current	8,545 (32%)	8,576 (32%)	8,462 (32%)	8,362 (31%)	8,385 (32%)	42,330 (32%)
Missing	24 (0%)	21 (0%)	25 (0%)	15 (0%)	25 (0%)	110 (0%)
**Alcohol intake**						
Never	5,082 (19%)	5,384 (20%)	5,245 (20%)	5,268 (20%)	5,124 (19%)	26,103 (20%)
Former	3,701 (14%)	3,571 (13%)	3,683 (14%)	3,605 (14%)	3,691 (14%)	18,251 (14%)
Current	17,847 (67%)	17,680 (66%)	17,699 (66%)	17,759 (67%)	17,818 (67%)	88,803 (67%)
Missing	12 (0%)	6 (0%)	14 (0%)	9 (0%)	9 (0%)	50 (0%)
**Physical measures**						
SBP, mmHg	127.0 (16.5)	127.1 (16.6)	127.3 (16.6)	127.2 (16.6)	127.6 (16.7)	127.2 (16.6)
DBP, mmHg	83.0 (10.2)	83.0 (10.1)	83.1 (10.1)	83.2 (10.2)	83.2 (10.3)	83.1 (10.2)
BMI, kg/m²	29.2 (5.1)	29.1 (5.1)	29.1 (5.1)	29.1 (5.0)	29.1 (5.0)	29.1 (5.1)
Waist-to-hip Ratio	0.90 (0.08)	0.90 (0.08)	0.90 (0.08)	0.90 (0.08)	0.90 (0.08)	0.90 (0.08)
**Laboratory measurements**						
HDL-C, mmol/L	1.00 (0.21)	1.00 (0.21)	1.00 (0.21)	1.00 (0.21)	1.00 (0.21)	1.00 (0.21)
LDL-C, mmol/L	2.45 (0.79)	2.47 (0.79)	2.46 (0.79)	2.46 (0.79)	2.45 (0.79)	2.46 (0.79)
Triglycerides, mmol/L	1.56 (0.65)	1.57 (0.65)	1.57 (0.66)	1.58 (0.66)	1.58 (0.66)	1.57 (0.66)
HbA1c, %	6.09 (1.70)	6.09 (1.70)	6.09 (1.71)	6.09 (1.70)	6.12 (1.75)	6.10 (1.71)
eGFR, ml/min/1.73m²*	101.7 (16.1)	101.6 (16.2)	101.7 (15.9)	101.6 (15.9)	101.6 (16.1)	101.7 (16.0)
**Prior disease** †						
Coronary heart disease	375 (1%)	380 (1%)	346 (1%)	386 (1%)	414 (2%)	1,901 (1%)
Stroke	249 (1%)	292 (1%)	295 (1%)	287 (1%)	291 (1%)	1,414 (1%)
Cancer	335 (1%)	318 (1%)	317 (1%)	315 (1%)	295 (1%)	1,580 (1%)
Diabetes ‡	4,955 (19%)	4,901 (18%)	4,900 (18%)	4,938 (19%)	5,102 (19%)	24,796 (19%)
Other §	2,197 (8%)	2,204 (8%)	2,166 (8%)	2,282 (9%)	2,264 (8%)	11,113 (8%)

Numbers are n (%) or mean (SD). SBP=systolic blood pressure, DBP=diastolic blood pressure, BMI=body mass index, HDL-C=high density lipoprotein cholesterol, LDL-C=low density lipoprotein cholesterol, HbA1c=glycosylated haemoglobin A1c

* Calculated using the 2021 CKD-EPI equation, based on NMR-measured creatinine levels.

† Self-reported previous diagnoses unless otherwise stated.

‡ Self-reported previously-diagnosed diabetes or glycosylated haemoglobin ≥6.5%.

§ Other diseases include self-reported emphysema, chronic kidney disease, peptic ulcer, liver cirrhosis, and peripheral arterial disease.

Webtable 5: Baseline characteristics of 133,207 participants aged 35-79 by fifth of PRS Oni-Orisan *et al.*

**Oni-Orisan et al/PGS004595/ European/141 SNPs**	**Fifth of the PRS distribution**					**All n=133,207**
	**1 n=26,642 (20%)**	**2 n=26,641 (20%)**	**3 n=26,641 (20%)**	**4 n=26,641 (20%)**	**5 n=26,642 (20%)**	
**Age, years**	51.3 (11.9)	51.3 (11.8)	51.2 (11.8)	51.1 (11.8)	51.3 (11.8)	51.2 (11.8)
**Resident of Coyoacán**	9,883 (37%)	10,000 (38%)	10,221 (38%)	10,280 (39%)	10,631 (40%)	51,015 (38%)
**Ancestry admixture percentage**						
Indigenous American	69.0 (17.5)	68.4 (17.8)	67.5 (17.8)	66.2 (17.9)	63.1 (17.7)	66.8 (17.9)
African	3.2 (2.6)	3.3 (2.7)	3.4 (2.8)	3.5 (2.9)	3.8 (3.0)	3.4 (2.8)
East Asian	1.4 (1.5)	1.4 (1.5)	1.4 (1.4)	1.4 (2.1)	1.5 (2.3)	1.4 (1.8)
European	26.4 (15.8)	26.9 (16.1)	27.7 (16.0)	28.8 (16.2)	31.6 (16.1)	28.3 (16.2)
**Highest attained educational level**						
University/high school	3,932 (15%)	4,007 (15%)	4,029 (15%)	4,224 (16%)	4,517 (17%)	20,709 (16%)
Middle school	6,629 (25%)	6,577 (25%)	6,696 (25%)	6,659 (25%)	6,871 (26%)	33,432 (25%)
Elementary	12,677 (48%)	12,718 (48%)	12,582 (47%)	12,651 (48%)	12,279 (46%)	62,907 (47%)
Other	3,391 (13%)	3,323 (12%)	3,320 (12%)	3,094 (12%)	2,963 (11%)	16,091 (12%)
Missing	13 (0%)	16 (0%)	14 (0%)	13 (0%)	12 (0%)	68 (0%)
**Smoking status**						
Never	13,096 (49%)	13,115 (49%)	13,046 (49%)	12,857 (48%)	12,477 (47%)	64,591 (48%)
Former	5,300 (20%)	5,223 (20%)	5,202 (20%)	5,164 (19%)	5,287 (20%)	26,176 (20%)
Current	8,232 (31%)	8,285 (31%)	8,368 (31%)	8,593 (32%)	8,852 (33%)	42,330 (32%)
Missing	14 (0%)	18 (0%)	25 (0%)	27 (0%)	26 (0%)	110 (0%)
**Alcohol intake**						
Never	5,202 (20%)	5,271 (20%)	5,261 (20%)	5,325 (20%)	5,044 (19%)	26,103 (20%)
Former	3,681 (14%)	3,747 (14%)	3,625 (14%)	3,563 (13%)	3,635 (14%)	18,251 (14%)
Current	17,745 (67%)	17,614 (66%)	17,744 (67%)	17,748 (67%)	17,952 (67%)	88,803 (67%)
Missing	14 (0%)	9 (0%)	11 (0%)	5 (0%)	11 (0%)	50 (0%)
**Physical measures**						
SBP, mmHg	126.6 (16.6)	127.0 (16.6)	127.4 (16.6)	127.3 (16.5)	127.8 (16.6)	127.2 (16.6)
DBP, mmHg	82.8 (10.2)	83.0 (10.1)	83.2 (10.3)	83.2 (10.2)	83.4 (10.2)	83.1 (10.2)
BMI, kg/m²	29.2 (5.1)	29.2 (5.1)	29.1 (5.1)	29.1 (5.1)	29.0 (5.0)	29.1 (5.1)
Waist-to-hip Ratio	0.90 (0.08)	0.90 (0.08)	0.90 (0.08)	0.90 (0.08)	0.90 (0.08)	0.90 (0.08)
**Laboratory measurements**						
HDL-C, mmol/L	1.00 (0.21)	1.00 (0.21)	1.00 (0.21)	1.00 (0.21)	1.00 (0.21)	1.00 (0.21)
LDL-C, mmol/L	2.43 (0.78)	2.45 (0.79)	2.45 (0.79)	2.47 (0.79)	2.49 (0.80)	2.46 (0.79)
Triglycerides, mmol/L	1.56 (0.65)	1.57 (0.66)	1.57 (0.66)	1.58 (0.66)	1.59 (0.67)	1.57 (0.66)
HbA1c, %	6.09 (1.69)	6.12 (1.73)	6.10 (1.72)	6.09 (1.72)	6.08 (1.70)	6.10 (1.71)
eGFR, ml/min/1.73m²*	101.9 (16.1)	101.8 (16.0)	101.6 (16.3)	101.6 (16.0)	101.4 (15.8)	101.7 (16.0)
**Prior disease** †						
Coronary heart disease	345 (1%)	306 (1%)	385 (1%)	396 (1%)	469 (2%)	1,901 (1%)
Stroke	259 (1%)	245 (1%)	322 (1%)	308 (1%)	280 (1%)	1,414 (1%)
Cancer	303 (1%)	326 (1%)	312 (1%)	320 (1%)	319 (1%)	1,580 (1%)
Diabetes ‡	4,903 (18%)	5,078 (19%)	5,027 (19%)	4,895 (18%)	4,893 (18%)	24,796 (19%)
Other §	2,202 (8%)	2,151 (8%)	2,309 (9%)	2,245 (8%)	2,206 (8%)	11,113 (8%)

Numbers are n (%) or mean (SD). SBP=systolic blood pressure, DBP=diastolic blood pressure, BMI=body mass index, HDL-C=high density lipoprotein cholesterol, LDL-C=low density lipoprotein cholesterol, HbA1c=glycosylated haemoglobin A1c

* Calculated using the 2021 CKD-EPI equation, based on NMR-measured creatinine levels.

† Self-reported previous diagnoses unless otherwise stated.

‡ Self-reported previously-diagnosed diabetes or glycosylated haemoglobin ≥6.5%.

§ Other diseases include self-reported emphysema, chronic kidney disease, peptic ulcer, liver cirrhosis, and peripheral arterial disease.

Webtable 6: Baseline characteristics of 133,207 participants aged 35-79 by fifth of PRS Koyama *et al.*

**Koyama et al/PGS000337/ Japanese and European/64,185 SNPs**	**Fifth of the PRS distribution**					**All n=133,207**
	**1 n=26,642 (20%)**	**2 n=26,641 (20%)**	**3 n=26,641 (20%)**	**4 n=26,641 (20%)**	**5 n=26,642 (20%)**	
**Age, years**	51.7 (12.0)	51.4 (11.9)	51.2 (11.8)	51.0 (11.8)	50.9 (11.6)	51.2 (11.8)
**Resident of Coyoacán**	10,764 (40%)	10,244 (38%)	10,137 (38%)	10,027 (38%)	9,843 (37%)	51,015 (38%)
**Ancestry admixture percentage**						
Indigenous American	62.6 (18.2)	65.9 (17.8)	67.4 (17.7)	68.6 (17.5)	69.7 (17.3)	66.8 (17.9)
African	3.7 (2.7)	3.5 (2.8)	3.4 (2.7)	3.3 (2.8)	3.3 (3.0)	3.4 (2.8)
East Asian	1.4 (1.7)	1.4 (1.6)	1.4 (1.9)	1.4 (1.7)	1.4 (2.1)	1.4 (1.8)
European	32.3 (16.7)	29.2 (16.2)	27.7 (16.0)	26.6 (15.7)	25.6 (15.4)	28.3 (16.2)
**Highest attained educational level**						
University/high school	4,846 (18%)	4,238 (16%)	4,102 (15%)	3,910 (15%)	3,613 (14%)	20,709 (16%)
Middle school	6,753 (25%)	6,774 (25%)	6,733 (25%)	6,641 (25%)	6,531 (25%)	33,432 (25%)
Elementary	12,110 (45%)	12,472 (47%)	12,567 (47%)	12,775 (48%)	12,983 (49%)	62,907 (47%)
Other	2,921 (11%)	3,146 (12%)	3,225 (12%)	3,296 (12%)	3,503 (13%)	16,091 (12%)
Missing	12 (0%)	11 (0%)	14 (0%)	19 (0%)	12 (0%)	68 (0%)
**Smoking status**						
Never	12,947 (49%)	13,096 (49%)	12,831 (48%)	12,915 (49%)	12,802 (48%)	64,591 (48%)
Former	5,258 (20%)	5,191 (20%)	5,356 (20%)	5,162 (19%)	5,209 (20%)	26,176 (20%)
Current	8,419 (32%)	8,332 (31%)	8,429 (32%)	8,539 (32%)	8,611 (32%)	42,330 (32%)
Missing	18 (0%)	22 (0%)	25 (0%)	25 (0%)	20 (0%)	110 (0%)
**Alcohol intake**						
Never	5,023 (19%)	5,300 (20%)	5,166 (19%)	5,293 (20%)	5,321 (20%)	26,103 (20%)
Former	3,495 (13%)	3,583 (13%)	3,590 (13%)	3,732 (14%)	3,851 (14%)	18,251 (14%)
Current	18,115 (68%)	17,746 (67%)	17,876 (67%)	17,608 (66%)	17,458 (66%)	88,803 (67%)
Missing	9 (0%)	12 (0%)	9 (0%)	8 (0%)	12 (0%)	50 (0%)
**Physical measures**						
SBP, mmHg	126.2 (16.2)	126.9 (16.6)	127.3 (16.5)	127.6 (16.8)	128.2 (16.9)	127.2 (16.6)
DBP, mmHg	82.5 (10.0)	82.9 (10.2)	83.1 (10.2)	83.3 (10.2)	83.6 (10.3)	83.1 (10.2)
BMI, kg/m^2	29.0 (5.1)	29.1 (5.1)	29.1 (5.0)	29.2 (5.1)	29.2 (5.1)	29.1 (5.1)
Waist-to-hip Ratio	0.90 (0.08)	0.90 (0.08)	0.90 (0.08)	0.90 (0.08)	0.90 (0.08)	0.90 (0.08)
**Laboratory measurements**						
HDL-C, mmol/L	1.01 (0.22)	1.01 (0.21)	1.00 (0.21)	1.00 (0.21)	0.99 (0.21)	1.00 (0.21)
LDL-C, mmol/L	2.45 (0.78)	2.45 (0.78)	2.46 (0.79)	2.46 (0.80)	2.46 (0.80)	2.46 (0.79)
Triglycerides, mmol/L	1.52 (0.63)	1.56 (0.65)	1.58 (0.66)	1.59 (0.66)	1.62 (0.68)	1.57 (0.66)
HbA1c, %	5.98 (1.58)	6.06 (1.66)	6.10 (1.72)	6.13 (1.75)	6.22 (1.83)	6.10 (1.71)
eGFR, ml/min/1.73m^2*	101.2 (15.9)	101.5 (16.0)	101.7 (16.1)	101.9 (16.1)	102.0 (16.0)	101.7 (16.0)
**Prior disease** †						
Coronary heart disease	325 (1%)	330 (1%)	357 (1%)	396 (1%)	493 (2%)	1,901 (1%)
Stroke	252 (1%)	279 (1%)	284 (1%)	263 (1%)	336 (1%)	1,414 (1%)
Cancer	336 (1%)	357 (1%)	285 (1%)	318 (1%)	284 (1%)	1,580 (1%)
Diabetes ‡	4,208 (16%)	4,731 (18%)	4,980 (19%)	5,164 (19%)	5,713 (21%)	24,796 (19%)
Other §	2,392 (9%)	2,242 (8%)	2,237 (8%)	2,151 (8%)	2,091 (8%)	11,113 (8%)

Numbers are n (%) or mean (SD). SBP=systolic blood pressure, DBP=diastolic blood pressure, BMI=body mass index, HDL-C=high density lipoprotein cholesterol, LDL-C=low density lipoprotein cholesterol, HbA1c=glycosylated haemoglobin A1c

* Calculated using the 2021 CKD-EPI equation, based on NMR-measured creatinine levels.

† Self-reported previous diagnoses unless otherwise stated.

‡ Self-reported previously-diagnosed diabetes or glycosylated haemoglobin ≥6.5%.

§ Other diseases include self-reported emphysema, chronic kidney disease, peptic ulcer, liver cirrhosis, and peripheral arterial disease.

Webtable 7: Baseline characteristics of 133,207 participants aged 35-79 by fifth of PRS Tcheandjieu *et al.*

**Tcheandjieu et al/PGS003446/ Multi-ancestry/485,464 SNPs**	**Fifth of the PRS distribution**					**All n=133,207**
	**1 n=26,642 (20%)**	**2 n=26,641 (20%)**	**3 n=26,641 (20%)**	**4 n=26,641 (20%)**	**5 n=26,642 (20%)**	
**Age, years**	51.0 (11.8)	51.0 (11.8)	51.0 (11.8)	51.3 (11.8)	51.9 (12.0)	51.2 (11.8)
**Resident of Coyoacán**	8,908 (33%)	9,590 (36%)	10,094 (38%)	10,650 (40%)	11,773 (44%)	51,015 (38%)
**Ancestry admixture percentage**						
Indigenous American	80.2 (14.4)	73.0 (15.4)	67.0 (15.5)	61.3 (15.2)	52.7 (15.4)	66.8 (17.9)
African	2.4 (2.9)	3.0 (2.8)	3.5 (2.6)	3.9 (2.6)	4.4 (2.6)	3.4 (2.8)
East Asian	1.3 (2.4)	1.4 (1.9)	1.5 (2.0)	1.5 (1.3)	1.5 (1.3)	1.4 (1.8)
European	16.1 (12.2)	22.6 (13.5)	28.0 (13.8)	33.4 (13.9)	41.3 (14.8)	28.3 (16.2)
**Highest attained educational level**						
University/high school	3,238 (12%)	3,697 (14%)	4,117 (15%)	4,400 (17%)	5,257 (20%)	20,709 (16%)
Middle school	6,051 (23%)	6,469 (24%)	6,823 (26%)	7,013 (26%)	7,076 (27%)	33,432 (25%)
Elementary	13,386 (50%)	13,054 (49%)	12,564 (47%)	12,299 (46%)	11,604 (44%)	62,907 (47%)
Other	3,951 (15%)	3,411 (13%)	3,121 (12%)	2,915 (11%)	2,693 (10%)	16,091 (12%)
Missing	16 (0%)	10 (0%)	16 (0%)	14 (0%)	12 (0%)	68 (0%)
**Smoking status**						
Never	14,516 (55%)	13,488 (51%)	12,956 (49%)	12,215 (46%)	11,416 (43%)	64,591 (48%)
Former	5,093 (19%)	5,151 (19%)	5,305 (20%)	5,260 (20%)	5,367 (20%)	26,176 (20%)
Current	7,011 (26%)	7,984 (30%)	8,359 (31%)	9,145 (34%)	9,831 (37%)	42,330 (32%)
Missing	22 (0%)	18 (0%)	21 (0%)	21 (0%)	28 (0%)	110 (0%)
**Alcohol intake**						
Never	5,649 (21%)	5,233 (20%)	5,163 (19%)	5,127 (19%)	4,931 (19%)	26,103 (20%)
Former	3,658 (14%)	3,712 (14%)	3,668 (14%)	3,548 (13%)	3,665 (14%)	18,251 (14%)
Current	17,330 (65%)	17,685 (66%)	17,799 (67%)	17,955 (67%)	18,034 (68%)	88,803 (67%)
Missing	5 (0%)	11 (0%)	11 (0%)	11 (0%)	12 (0%)	50 (0%)
**Physical measures**						
SBP, mmHg	126.2 (16.2)	126.8 (16.4)	127.3 (16.6)	127.6 (16.7)	128.3 (17.0)	127.2 (16.6)
DBP, mmHg	82.5 (9.9)	82.9 (10.1)	83.2 (10.2)	83.3 (10.3)	83.6 (10.4)	83.1 (10.2)
BMI, kg/m²	29.0 (4.9)	29.1 (5.0)	29.2 (5.0)	29.2 (5.1)	29.1 (5.3)	29.1 (5.1)
Waist-to-hip Ratio	0.90 (0.07)	0.90 (0.08)	0.90 (0.08)	0.90 (0.08)	0.90 (0.08)	0.90 (0.08)
**Laboratory measurements**						
HDL-C, mmol/L	1.00 (0.21)	1.00 (0.21)	1.00 (0.21)	1.00 (0.21)	1.00 (0.22)	1.00 (0.21)
LDL-C, mmol/L	2.39 (0.77)	2.43 (0.78)	2.45 (0.78)	2.48 (0.78)	2.53 (0.82)	2.46 (0.79)
Triglycerides, mmol/L	1.57 (0.64)	1.57 (0.66)	1.58 (0.66)	1.57 (0.65)	1.58 (0.67)	1.57 (0.66)
HbA1c, %	6.16 (1.77)	6.12 (1.72)	6.10 (1.71)	6.08 (1.71)	6.04 (1.66)	6.10 (1.71)
eGFR, ml/min/1.73m²*	102.9 (15.6)	102.4 (15.9)	101.8 (16.0)	101.2 (16.1)	99.9 (16.4)	101.7 (16.0)
**Prior disease** †						
Coronary heart disease	249 (1%)	275 (1%)	355 (1%)	450 (2%)	572 (2%)	1,901 (1%)
Stroke	267 (1%)	289 (1%)	269 (1%)	276 (1%)	313 (1%)	1,414 (1%)
Cancer	278 (1%)	295 (1%)	282 (1%)	335 (1%)	390 (1%)	1,580 (1%)
Diabetes ‡	5,066 (19%)	5,053 (19%)	5,033 (19%)	4,816 (18%)	4,828 (18%)	24,796 (19%)
Other §	1,880 (7%)	2,106 (8%)	2,180 (8%)	2,346 (9%)	2,601 (10%)	11,113 (8%)

Numbers are n (%) or mean (SD). SBP=systolic blood pressure, DBP=diastolic blood pressure, BMI=body mass index, HDL-C=high density lipoprotein cholesterol, LDL-C=low density lipoprotein cholesterol, HbA1c=glycosylated haemoglobin A1c

* Calculated using the 2021 CKD-EPI equation, based on NMR-measured creatinine levels.

† Self-reported previous diagnoses unless otherwise stated.

‡ Self-reported previously-diagnosed diabetes or glycosylated haemoglobin ≥6.5%.

§ Other diseases include self-reported emphysema, chronic kidney disease, peptic ulcer, liver cirrhosis, and peripheral arterial disease.

Webtable 8: Baseline characteristics of 133,207 participants aged 35-79 by fifth of PRS Tamlander *et al.*

**Tamlander et al/ PGS001780/ European/1,087,958 SNPs**	**Fifth of the PRS distribution**					**All n=133,207**
	**1 n=26,642 (20%)**	**2 n=26,641 (20%)**	**3 n=26,641 (20%)**	**4 n=26,641 (20%)**	**5 n=26,642 (20%)**	
**Age, years**	52.0 (12.0)	51.4 (11.9)	51.0 (11.8)	51.0 (11.8)	50.7 (11.7)	51.2 (11.8)
**Resident of Coyoacán**	11,363 (43%)	10,657 (40%)	10,099 (38%)	9,634 (36%)	9,262 (35%)	51,015 (38%)
**Ancestry admixture percentage**						
Indigenous American	56.7 (16.9)	63.7 (17.0)	67.6 (17.0)	71.1 (16.8)	75.1 (15.9)	66.8 (17.9)
African	4.3 (3.1)	3.8 (2.8)	3.4 (2.7)	3.1 (2.6)	2.6 (2.4)	3.4 (2.8)
East Asian	1.5 (1.5)	1.4 (1.7)	1.4 (1.8)	1.4 (2.0)	1.4 (2.1)	1.4 (1.8)
European	37.5 (15.8)	31.1 (15.5)	27.5 (15.3)	24.5 (14.9)	20.9 (14.1)	28.3 (16.2)
**Highest attained educational level**						
University/high school	5,318 (20%)	4,523 (17%)	4,002 (15%)	3,605 (14%)	3,261 (12%)	20,709 (16%)
Middle school	7,075 (27%)	6,861 (26%)	6,787 (25%)	6,478 (24%)	6,231 (23%)	33,432 (25%)
Elementary	11,578 (43%)	12,270 (46%)	12,655 (48%)	13,087 (49%)	13,317 (50%)	62,907 (47%)
Other	2,654 (10%)	2,975 (11%)	3,190 (12%)	3,459 (13%)	3,813 (14%)	16,091 (12%)
Missing	17 (0%)	12 (0%)	7 (0%)	12 (0%)	20 (0%)	68 (0%)
**Smoking status**						
Never	12,389 (47%)	12,766 (48%)	12,950 (49%)	13,056 (49%)	13,430 (50%)	64,591 (48%)
Former	5,382 (20%)	5,189 (19%)	5,207 (20%)	5,305 (20%)	5,093 (19%)	26,176 (20%)
Current	8,846 (33%)	8,661 (33%)	8,464 (32%)	8,259 (31%)	8,100 (30%)	42,330 (32%)
Missing	25 (0%)	25 (0%)	20 (0%)	21 (0%)	19 (0%)	110 (0%)
**Alcohol intake**						
Never	4,887 (18%)	5,091 (19%)	5,212 (20%)	5,389 (20%)	5,524 (21%)	26,103 (20%)
Former	3,494 (13%)	3,527 (13%)	3,621 (14%)	3,769 (14%)	3,840 (14%)	18,251 (14%)
Current	18,249 (69%)	18,012 (68%)	17,800 (67%)	17,477 (66%)	17,265 (65%)	88,803 (67%)
Missing	12 (0%)	11 (0%)	8 (0%)	6 (0%)	13 (0%)	50 (0%)
**Physical measures**						
SBP, mmHg	126.7 (16.4)	127.1 (16.4)	127.0 (16.6)	127.6 (16.7)	127.9 (16.8)	127.2 (16.6)
DBP, mmHg	82.9 (10.1)	83.0 (10.2)	83.0 (10.2)	83.3 (10.2)	83.4 (10.2)	83.1 (10.2)
BMI, kg/m²	29.0 (5.2)	29.1 (5.1)	29.1 (5.1)	29.2 (5.0)	29.2 (5.0)	29.1 (5.1)
Waist-to-hip Ratio	0.90 (0.08)	0.90 (0.08)	0.90 (0.08)	0.90 (0.08)	0.91 (0.07)	0.90 (0.08)
**Laboratory measurements**						
HDL-C, mmol/L	1.01 (0.22)	1.00 (0.21)	1.00 (0.21)	1.00 (0.21)	0.99 (0.21)	1.00 (0.21)
LDL-C, mmol/L	2.47 (0.78)	2.47 (0.78)	2.46 (0.79)	2.45 (0.80)	2.45 (0.80)	2.46 (0.79)
Triglycerides, mmol/L	1.53 (0.64)	1.56 (0.65)	1.57 (0.66)	1.59 (0.66)	1.63 (0.68)	1.57 (0.66)
HbA1c, %	5.95 (1.55)	6.03 (1.63)	6.10 (1.72)	6.15 (1.76)	6.26 (1.87)	6.10 (1.71)
eGFR, ml/min/1.73m²*	100.5 (16.0)	101.4 (16.0)	101.8 (16.0)	102.1 (16.1)	102.4 (16.0)	101.7 (16.0)
**Prior disease** †						
Coronary heart disease	379 (1%)	367 (1%)	357 (1%)	384 (1%)	414 (2%)	1,901 (1%)
Stroke	255 (1%)	279 (1%)	300 (1%)	278 (1%)	302 (1%)	1,414 (1%)
Cancer	367 (1%)	329 (1%)	301 (1%)	308 (1%)	275 (1%)	1,580 (1%)
Diabetes ‡	4,096 (15%)	4,624 (17%)	4,952 (19%)	5,287 (20%)	5,837 (22%)	24,796 (19%)
Other §	2,504 (9%)	2,352 (9%)	2,233 (8%)	2,135 (8%)	1,889 (7%)	11,113 (8%)

Numbers are n (%) or mean (SD). SBP=systolic blood pressure, DBP=diastolic blood pressure, BMI=body mass index, HDL-C=high density lipoprotein cholesterol, LDL-C=low density lipoprotein cholesterol, HbA1c=glycosylated haemoglobin A1c

* Calculated using the 2021 CKD-EPI equation, based on NMR-measured creatinine levels.

† Self-reported previous diagnoses unless otherwise stated.

‡ Self-reported previously-diagnosed diabetes or glycosylated haemoglobin ≥6.5%.

§ Other diseases include self-reported emphysema, chronic kidney disease, peptic ulcer, liver cirrhosis, and peripheral arterial disease.

Webtable 9: Baseline characteristics of 133,207 participants aged 35-79 by fifth of PRS Patel *et al.*

**Patel et al/PGS003725/ Multi-ancestry/1,273,824 SNPs**	**Fifth of the PRS distribution**					**All n=133,207**
	**1 n=26,642 (20%)**	**2 n=26,641 (20%)**	**3 n=26,641 (20%)**	**4 n=26,641 (20%)**	**5 n=26,642 (20%)**	
**Age, years**	51.5 (12.0)	51.4 (11.9)	51.3 (11.9)	51.1 (11.7)	50.9 (11.6)	51.2 (11.8)
**Resident of Coyoacán**	9,980 (37%)	10,079 (38%)	10,204 (38%)	10,162 (38%)	10,590 (40%)	51,015 (38%)
**Ancestry admixture percentage**						
Indigenous American	70.4 (17.7)	68.8 (17.7)	67.5 (17.7)	65.6 (17.7)	62.0 (17.4)	66.8 (17.9)
African	2.9 (2.3)	3.2 (2.5)	3.4 (2.7)	3.6 (2.8)	4.1 (3.4)	3.4 (2.8)
East Asian	1.3 (1.5)	1.4 (1.7)	1.4 (1.9)	1.4 (1.7)	1.5 (2.3)	1.4 (1.8)
European	25.4 (16.0)	26.7 (16.1)	27.7 (16.0)	29.4 (16.0)	32.3 (15.9)	28.3 (16.2)
**Highest attained educational level**						
University/high school	4,224 (16%)	4,076 (15%)	4,110 (15%)	4,080 (15%)	4,219 (16%)	20,709 (16%)
Middle school	6,441 (24%)	6,761 (25%)	6,658 (25%)	6,691 (25%)	6,881 (26%)	33,432 (25%)
Elementary	12,709 (48%)	12,498 (47%)	12,576 (47%)	12,642 (47%)	12,482 (47%)	62,907 (47%)
Other	3,256 (12%)	3,292 (12%)	3,278 (12%)	3,218 (12%)	3,047 (11%)	16,091 (12%)
Missing	12 (0%)	14 (0%)	19 (0%)	10 (0%)	13 (0%)	68 (0%)
**Smoking status**						
Never	13,720 (52%)	13,227 (50%)	12,869 (48%)	12,634 (47%)	12,141 (46%)	64,591 (48%)
Former	5,166 (19%)	5,245 (20%)	5,302 (20%)	5,224 (20%)	5,239 (20%)	26,176 (20%)
Current	7,732 (29%)	8,149 (31%)	8,450 (32%)	8,758 (33%)	9,241 (35%)	42,330 (32%)
Missing	24 (0%)	20 (0%)	20 (0%)	25 (0%)	21 (0%)	110 (0%)
**Alcohol intake**						
Never	5,127 (19%)	5,196 (20%)	5,336 (20%)	5,331 (20%)	5,113 (19%)	26,103 (20%)
Former	3,616 (14%)	3,599 (14%)	3,608 (14%)	3,688 (14%)	3,740 (14%)	18,251 (14%)
Current	17,890 (67%)	17,834 (67%)	17,690 (66%)	17,611 (66%)	17,778 (67%)	88,803 (67%)
Missing	9 (0%)	12 (0%)	7 (0%)	11 (0%)	11 (0%)	50 (0%)
**Physical measures**						
SBP, mmHg	125.8 (16.1)	126.6 (16.4)	127.2 (16.4)	127.9 (16.9)	128.6 (17.0)	127.2 (16.6)
DBP, mmHg	82.3 (10.1)	82.7 (10.1)	83.2 (10.1)	83.4 (10.3)	83.9 (10.4)	83.1 (10.2)
BMI, kg/m²	28.9 (5.0)	29.1 (5.0)	29.1 (5.1)	29.2 (5.1)	29.3 (5.2)	29.1 (5.1)
Waist-to-hip Ratio	0.90 (0.08)	0.90 (0.08)	0.90 (0.08)	0.90 (0.08)	0.90 (0.08)	0.90 (0.08)
**Laboratory measurements**						
HDL-C, mmol/L	1.01 (0.22)	1.01 (0.21)	1.00 (0.21)	1.00 (0.21)	0.99 (0.21)	1.00 (0.21)
LDL-C, mmol/L	2.40 (0.76)	2.44 (0.78)	2.46 (0.79)	2.48 (0.80)	2.51 (0.82)	2.46 (0.79)
Triglycerides, mmol/L	1.50 (0.61)	1.55 (0.64)	1.58 (0.66)	1.60 (0.68)	1.63 (0.70)	1.57 (0.66)
HbA1c, %	5.98 (1.58)	6.05 (1.64)	6.09 (1.69)	6.16 (1.78)	6.22 (1.84)	6.10 (1.71)
eGFR, ml/min/1.73m²*	102.0 (15.7)	101.9 (15.8)	101.5 (16.2)	101.6 (16.1)	101.3 (16.4)	101.7 (16.0)
**Prior disease** †						
Coronary heart disease	285 (1%)	324 (1%)	332 (1%)	389 (1%)	571 (2%)	1,901 (1%)
Stroke	233 (1%)	259 (1%)	310 (1%)	280 (1%)	332 (1%)	1,414 (1%)
Cancer	312 (1%)	321 (1%)	316 (1%)	319 (1%)	312 (1%)	1,580 (1%)
Diabetes ‡	4,195 (16%)	4,583 (17%)	4,922 (18%)	5,353 (20%)	5,743 (22%)	24,796 (19%)
Other §	2,253 (8%)	2,208 (8%)	2,193 (8%)	2,269 (9%)	2,190 (8%)	11,113 (8%)

Numbers are n (%) or mean (SD). SBP=systolic blood pressure, DBP=diastolic blood pressure, BMI=body mass index, HDL-C=high density lipoprotein cholesterol, LDL-C=low density lipoprotein cholesterol, HbA1c=glycosylated haemoglobin A1c

* Calculated using the 2021 CKD-EPI equation, based on NMR-measured creatinine levels.

† Self-reported previous diagnoses unless otherwise stated.

‡ Self-reported previously-diagnosed diabetes or glycosylated haemoglobin ≥6.5%.

§ Other diseases include self-reported emphysema, chronic kidney disease, peptic ulcer, liver cirrhosis, and peripheral arterial disease.

Webtable 10: Baseline characteristics of 133,207 participants aged 35-79 by fifth of PRS Inouye *et al.*

**Inouye et al/PGS000018/ European/1,720,068 SNPs**	**Fifth of the PRS distribution**					**All n=133,207**
	**1 n=26,642 (20%)**	**2 n=26,641 (20%)**	**3 n=26,641 (20%)**	**4 n=26,641 (20%)**	**5 n=26,642 (20%)**	
**Age, years**	52.4 (12.1)	51.4 (11.8)	51.0 (11.8)	50.8 (11.8)	50.7 (11.7)	51.2 (11.8)
**Resident of Coyoacán**	11,967 (45%)	10,593 (40%)	10,046 (38%)	9,484 (36%)	8,925 (33%)	51,015 (38%)
**Ancestry admixture percentage**						
Indigenous American	51.8 (15.1)	61.7 (15.2)	67.8 (15.6)	73.3 (15.3)	79.6 (14.3)	66.8 (17.9)
African	4.6 (2.8)	4.0 (2.8)	3.5 (2.8)	2.9 (2.6)	2.3 (2.3)	3.4 (2.8)
East Asian	1.5 (1.5)	1.5 (1.6)	1.5 (1.9)	1.4 (1.8)	1.3 (2.2)	1.4 (1.8)
European	42.1 (14.4)	32.8 (13.8)	27.3 (13.9)	22.4 (13.5)	16.9 (12.4)	28.3 (16.2)
**Highest attained educational level**						
University/high school	5,825 (22%)	4,492 (17%)	4,008 (15%)	3,480 (13%)	2,904 (11%)	20,709 (16%)
Middle school	7,149 (27%)	7,077 (27%)	6,792 (26%)	6,366 (24%)	6,048 (23%)	33,432 (25%)
Elementary	11,220 (42%)	12,188 (46%)	12,757 (48%)	13,209 (50%)	13,533 (51%)	62,907 (47%)
Other	2,440 (9%)	2,872 (11%)	3,072 (12%)	3,568 (13%)	4,139 (16%)	16,091 (12%)
Missing	8 (0%)	12 (0%)	12 (0%)	18 (0%)	18 (0%)	68 (0%)
**Smoking status**						
Never	11,982 (45%)	12,541 (47%)	12,966 (49%)	13,351 (50%)	13,751 (52%)	64,591 (48%)
Former	5,364 (20%)	5,314 (20%)	5,198 (20%)	5,200 (20%)	5,100 (19%)	26,176 (20%)
Current	9,268 (35%)	8,771 (33%)	8,454 (32%)	8,067 (30%)	7,770 (29%)	42,330 (32%)
Missing	28 (0%)	15 (0%)	23 (0%)	23 (0%)	21 (0%)	110 (0%)
**Alcohol intake**						
Never	4,730 (18%)	5,224 (20%)	5,215 (20%)	5,404 (20%)	5,530 (21%)	26,103 (20%)
Former	3,379 (13%)	3,567 (13%)	3,661 (14%)	3,708 (14%)	3,936 (15%)	18,251 (14%)
Current	18,520 (70%)	17,840 (67%)	17,757 (67%)	17,519 (66%)	17,167 (64%)	88,803 (67%)
Missing	13 (0%)	10 (0%)	8 (0%)	10 (0%)	9 (0%)	50 (0%)
**Physical measures**						
SBP, mmHg	126.9 (16.5)	127.1 (16.4)	127.0 (16.6)	127.5 (16.8)	127.7 (16.7)	127.2 (16.6)
DBP, mmHg	83.0 (10.1)	83.1 (10.2)	83.1 (10.2)	83.2 (10.2)	83.3 (10.2)	83.1 (10.2)
BMI, kg/m²	28.9 (5.2)	29.1 (5.1)	29.1 (5.1)	29.2 (5.0)	29.2 (4.9)	29.1 (5.1)
Waist-to-hip Ratio	0.90 (0.08)	0.90 (0.08)	0.90 (0.08)	0.90 (0.08)	0.91 (0.07)	0.90 (0.08)
**Laboratory measurements**						
HDL-C, mmol/L	1.01 (0.22)	1.00 (0.21)	1.00 (0.21)	1.00 (0.21)	0.99 (0.21)	1.00 (0.21)
LDL-C, mmol/L	2.50 (0.78)	2.48 (0.80)	2.45 (0.79)	2.44 (0.78)	2.42 (0.79)	2.46 (0.79)
Triglycerides, mmol/L	1.52 (0.63)	1.56 (0.66)	1.57 (0.66)	1.60 (0.67)	1.62 (0.67)	1.57 (0.66)
HbA1c, %	5.90 (1.48)	6.04 (1.66)	6.10 (1.72)	6.16 (1.76)	6.28 (1.89)	6.10 (1.71)
eGFR, ml/min/1.73m²*	99.8 (16.1)	101.2 (16.0)	102.0 (15.9)	102.3 (16.1)	103.0 (15.9)	101.7 (16.0)
**Prior disease †**						
Coronary heart disease	432 (2%)	382 (1%)	364 (1%)	332 (1%)	391 (1%)	1,901 (1%)
Stroke	255 (1%)	267 (1%)	298 (1%)	285 (1%)	309 (1%)	1,414 (1%)
Cancer	384 (1%)	348 (1%)	318 (1%)	263 (1%)	267 (1%)	1,580 (1%)
Diabetes ‡	3,898 (15%)	4,589 (17%)	4,959 (19%)	5,418 (20%)	5,932 (22%)	24,796 (19%)
Other §	2,664 (10%)	2,319 (9%)	2,231 (8%)	2,029 (8%)	1,870 (7%)	11,113 (8%)

Numbers are n (%) or mean (SD). SBP=systolic blood pressure, DBP=diastolic blood pressure, BMI=body mass index, HDL-C=high density lipoprotein cholesterol, LDL-C=low density lipoprotein cholesterol, HbA1c=glycosylated haemoglobin A1c

* Calculated using the 2021 CKD-EPI equation, based on NMR-measured creatinine levels.

† Self-reported previous diagnoses unless otherwise stated.

‡ Self-reported previously-diagnosed diabetes or glycosylated haemoglobin ≥6.5%.

§ Other diseases include self-reported emphysema, chronic kidney disease, peptic ulcer, liver cirrhosis, and peripheral arterial disease.

Webtable 11: Baseline characteristics of 133,207 participants aged 35-79 by fifth of PRS Khera *et al.*

**Khera et al/PGS000013/ European/6,472,620 SNPs**	**Fifth of the PRS distribution**					**All n=133,207**
	**1 n=26,642 (20%)**	**2 n=26,641 (20%)**	**3 n=26,641 (20%)**	**4 n=26,641 (20%)**	**5 n=26,642 (20%)**	
**Age, years**	51.4 (11.9)	51.2 (11.8)	51.3 (11.8)	51.1 (11.8)	51.2 (11.8)	51.2 (11.8)
**Resident of Coyoacán**	10,270 (39%)	10,129 (38%)	10,096 (38%)	10,227 (38%)	10,293 (39%)	51,015 (38%)
**Ancestry admixture percentage**						
Indigenous American	66.1 (17.7)	67.9 (17.9)	67.9 (17.8)	67.2 (17.9)	65.1 (17.8)	66.8 (17.9)
African	3.7 (3.4)	3.3 (2.7)	3.3 (2.6)	3.4 (2.6)	3.5 (2.6)	3.4 (2.8)
East Asian	1.4 (1.4)	1.4 (1.6)	1.4 (1.6)	1.4 (1.9)	1.5 (2.4)	1.4 (1.8)
European	28.7 (16.0)	27.4 (16.2)	27.4 (16.1)	28.0 (16.2)	29.9 (16.2)	28.3 (16.2)
**Highest attained educational level**						
University/high school	4,272 (16%)	4,068 (15%)	4,091 (15%)	4,108 (15%)	4,170 (16%)	20,709 (16%)
Middle school	6,707 (25%)	6,746 (25%)	6,642 (25%)	6,683 (25%)	6,654 (25%)	33,432 (25%)
Elementary	12,395 (47%)	12,676 (48%)	12,604 (47%)	12,657 (48%)	12,575 (47%)	62,907 (47%)
Other	3,251 (12%)	3,143 (12%)	3,291 (12%)	3,176 (12%)	3,230 (12%)	16,091 (12%)
Missing	17 (0%)	8 (0%)	13 (0%)	17 (0%)	13 (0%)	68 (0%)
**Smoking status**						
Never	13,064 (49%)	13,057 (49%)	13,048 (49%)	12,703 (48%)	12,719 (48%)	64,591 (48%)
Former	5,294 (20%)	5,132 (19%)	5,187 (19%)	5,256 (20%)	5,307 (20%)	26,176 (20%)
Current	8,264 (31%)	8,437 (32%)	8,382 (31%)	8,654 (33%)	8,593 (32%)	42,330 (32%)
Missing	20 (0%)	15 (0%)	24 (0%)	28 (0%)	23 (0%)	110 (0%)
**Alcohol intake**						
Never	5,175 (19%)	5,122 (19%)	5,258 (20%)	5,300 (20%)	5,248 (20%)	26,103 (20%)
Former	3,641 (14%)	3,617 (14%)	3,635 (14%)	3,659 (14%)	3,699 (14%)	18,251 (14%)
Current	17,815 (67%)	17,896 (67%)	17,737 (67%)	17,670 (66%)	17,685 (66%)	88,803 (67%)
Missing	11 (0%)	6 (0%)	11 (0%)	12 (0%)	10 (0%)	50 (0%)
**Physical measures**						
SBP, mmHg	126.7 (16.6)	126.9 (16.3)	127.3 (16.6)	127.3 (16.7)	128.0 (16.8)	127.2 (16.6)
DBP, mmHg	82.9 (10.1)	83.0 (10.2)	83.1 (10.2)	83.2 (10.2)	83.4 (10.3)	83.1 (10.2)
BMI, kg/m²	29.2 (5.2)	29.1 (5.0)	29.1 (5.0)	29.1 (5.1)	29.0 (5.1)	29.1 (5.1)
Waist-to-hip Ratio	0.90 (0.08)	0.90 (0.08)	0.90 (0.08)	0.90 (0.08)	0.90 (0.08)	0.90 (0.08)
**Laboratory measurements**						
HDL-C, mmol/L	1.00 (0.21)	1.00 (0.21)	1.00 (0.21)	1.00 (0.21)	1.00 (0.21)	1.00 (0.21)
LDL-C, mmol/L	2.42 (0.77)	2.44 (0.79)	2.45 (0.79)	2.46 (0.80)	2.51 (0.80)	2.46 (0.79)
Triglycerides, mmol/L	1.55 (0.64)	1.57 (0.65)	1.58 (0.67)	1.58 (0.66)	1.59 (0.67)	1.57 (0.66)
HbA1c, %	6.05 (1.66)	6.09 (1.70)	6.10 (1.71)	6.11 (1.73)	6.13 (1.76)	6.10 (1.71)
eGFR, ml/min/1.73m²*	101.5 (15.9)	101.8 (16.2)	101.6 (16.4)	101.9 (15.8)	101.5 (15.9)	101.7 (16.0)
**Prior disease** †						
Coronary heart disease	314 (1%)	334 (1%)	328 (1%)	414 (2%)	511 (2%)	1,901 (1%)
Stroke	261 (1%)	269 (1%)	302 (1%)	284 (1%)	298 (1%)	1,414 (1%)
Cancer	318 (1%)	326 (1%)	314 (1%)	304 (1%)	318 (1%)	1,580 (1%)
Diabetes ‡	4,600 (17%)	4,969 (19%)	4,966 (19%)	5,037 (19%)	5,224 (20%)	24,796 (19%)
Other §	2,308 (9%)	2,195 (8%)	2,200 (8%)	2,202 (8%)	2,208 (8%)	11,113 (8%)

Numbers are n (%) or mean (SD). SBP=systolic blood pressure, DBP=diastolic blood pressure, BMI=body mass index, HDL-C=high density lipoprotein cholesterol, LDL-C=low density lipoprotein cholesterol, HbA1c=glycosylated haemoglobin A1c

* Calculated using the 2021 CKD-EPI equation, based on NMR-measured creatinine levels.

† Self-reported previous diagnoses unless otherwise stated.

‡ Self-reported previously-diagnosed diabetes or glycosylated haemoglobin ≥6.5%.

§ Other diseases include self-reported emphysema, chronic kidney disease, peptic ulcer, liver cirrhosis, and peripheral arterial disease.

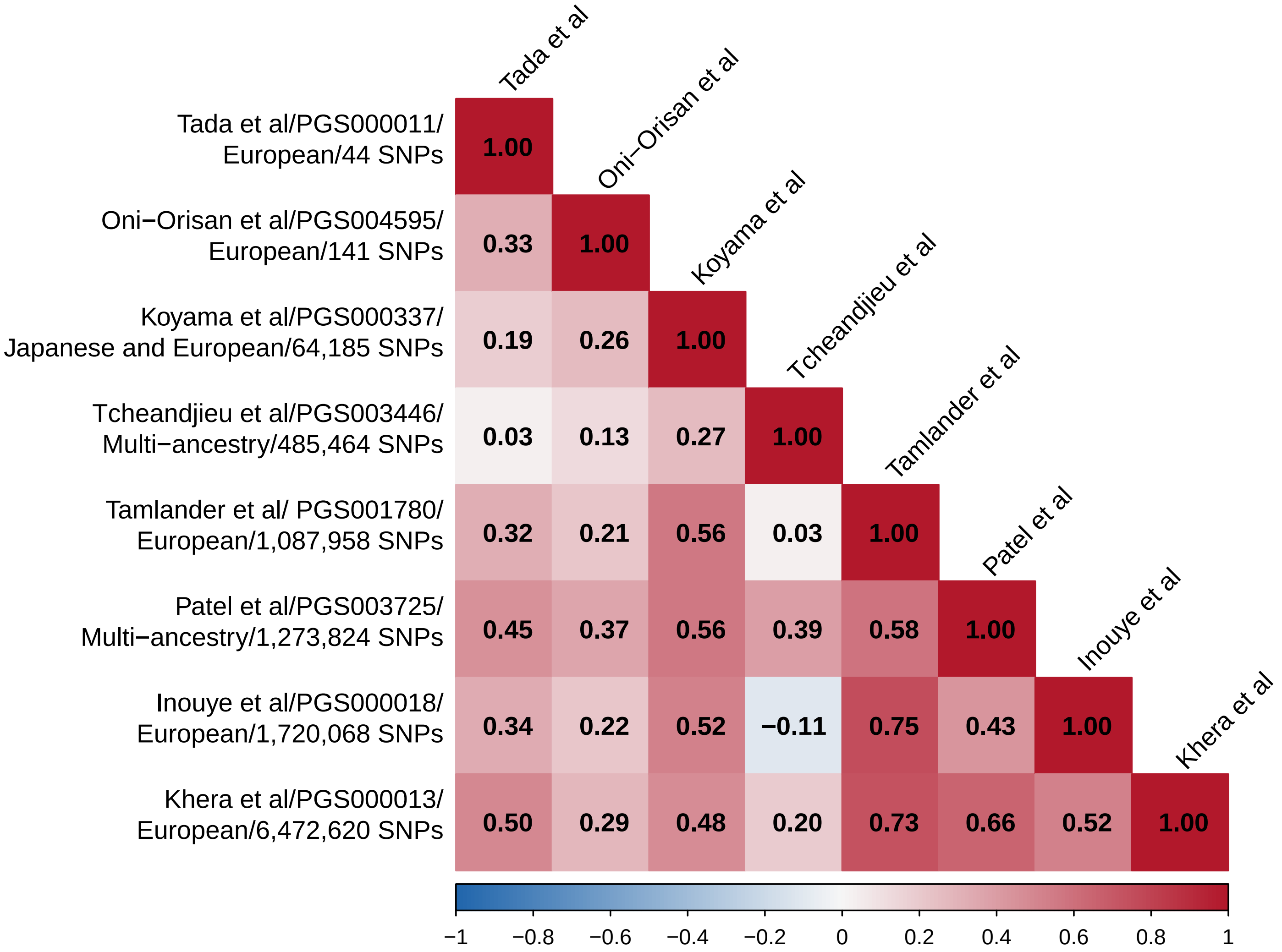

Webfigure 1: Pairwise correlations between the eight selected PRS

PRS / baseline age	CHD cases	CHD controls	OR per SD (95% CI)	AUC (95%CI)
**Tada et al/PGS000011/ European/44 SNPs**				
35-54 yrs	1,720	83,613	1.03 (0.98-1.08)	0.706 (0.694-0.719)
55-64 yrs	1,672	23,727	1.06 (1.00-1.11)	0.653 (0.639-0.667)
65-79 yrs	1,771	20,704	1.06 (1.01-1.11)	0.621 (0.608-0.635)
Overall	5,163	128,044	1.05 (1.02-1.08)	0.745 (0.739-0.752)
			Trend: X^2(df=1)=0.69 (p=0.41)	
			Heterogeneity:Q=0.91 (p=0.64)	
**Oni-Orisan et al/PGS004595/ European/141 SNPs**				
35-54 yrs	1,720	83,613	1.05 (1.00-1.11)	0.707 (0.694-0.719)
55-64 yrs	1,672	23,727	1.11 (1.06-1.17)	0.654 (0.640-0.668)
65-79 yrs	1,771	20,704	1.11 (1.06-1.17)	0.624 (0.610-0.638)
Overall	5,163	128,044	1.09 (1.06-1.13)	0.746 (0.739-0.752)
			Trend: X^2(df=1)=2.5 (p=0.11)	
			Heterogeneity:Q=3.3 (p=0.19)	
**Koyama et al/PGS000337/ Japanese and European/64,185 SNPs**				
35-54 yrs	1,720	83,613	1.20 (1.14-1.26)	0.710 (0.698-0.723)
55-64 yrs	1,672	23,727	1.16 (1.10-1.22)	0.656 (0.643-0.670)
65-79 yrs	1,771	20,704	1.21 (1.15-1.27)	0.629 (0.616-0.643)
Overall	5,163	128,044	1.20 (1.17-1.23)	0.748 (0.742-0.754)
			Trend: X^2(df=1)=0.05 (p=0.82)	
			Heterogeneity:Q=1.52 (p=0.47)	
**Tcheandjieu et al/PGS003446/ Multi-ancestry/485,464 SNPs**				
35-54 yrs	1,720	83,613	1.17 (1.10-1.24)	0.707 (0.695-0.720)
55-64 yrs	1,672	23,727	1.15 (1.09-1.23)	0.655 (0.641-0.668)
65-79 yrs	1,771	20,704	1.13 (1.06-1.20)	0.623 (0.609-0.637)
Overall	5,163	128,044	1.16 (1.12-1.20)	0.746 (0.740-0.753)
			Trend: X^2(df=1)=0.67 (p=0.41)	
			Heterogeneity:Q=0.67 (p=0.72)	
**Tamlander et al/ PGS001780/ European/1,087,958 SNPs**				
35-54 yrs	1,720	83,613	1.17 (1.11-1.23)	0.708 (0.696-0.721)
55-64 yrs	1,672	23,727	1.18 (1.12-1.25)	0.657 (0.644-0.671)
65-79 yrs	1,771	20,704	1.21 (1.15-1.28)	0.630 (0.616-0.644)
Overall	5,163	128,044	1.20 (1.16-1.23)	0.748 (0.741-0.754)
			Trend: X^2(df=1)=0.79 (p=0.37)	
			Heterogeneity:Q=0.85 (p=0.65)	
**Patel et al/PGS003725/ Multi-ancestry/1,273,824 SNPs**				
35-54 yrs	1,720	83,613	1.19 (1.14-1.25)	0.710 (0.697-0.722)
55-64 yrs	1,672	23,727	1.25 (1.19-1.32)	0.663 (0.649-0.676)
65-79 yrs	1,771	20,704	1.24 (1.18-1.30)	0.632 (0.618-0.646)
Overall	5,163	128,044	1.24 (1.20-1.28)	0.749 (0.743-0.755)
			Trend: X^2(df=1)=1.34 (p=0.25)	
			Heterogeneity:Q=2.15 (p=0.34)	
**Inouye et al/PGS000018/ European/1,720,068 SNPs**				
35-54 yrs	1,720	83,613	1.17 (1.11-1.24)	0.709 (0.696-0.721)
55-64 yrs	1,672	23,727	1.16 (1.09-1.23)	0.655 (0.642-0.669)
65-79 yrs	1,771	20,704	1.22 (1.15-1.30)	0.629 (0.615-0.643)
Overall	5,163	128,044	1.19 (1.15-1.23)	0.747 (0.741-0.753)
			Trend: X^2(df=1)=0.97 (p=0.32)	
			Heterogeneity:Q=1.61 (p=0.45)	
**Khera et al/PGS000013/ European/6,472,620 SNPs**				
35-54 yrs	1,720	83,613	1.13 (1.07-1.18)	0.708 (0.695-0.720)
55-64 yrs	1,672	23,727	1.17 (1.11-1.23)	0.658 (0.644-0.672)
65-79 yrs	1,771	20,704	1.20 (1.15-1.26)	0.630 (0.616-0.643)
Overall	5,163	128,044	1.17 (1.14-1.20)	0.747 (0.741-0.754)
			Trend: X^2(df=1)=2.95 (p=0.086)	
			Heterogeneity:Q=2.98 (p=0.23)	

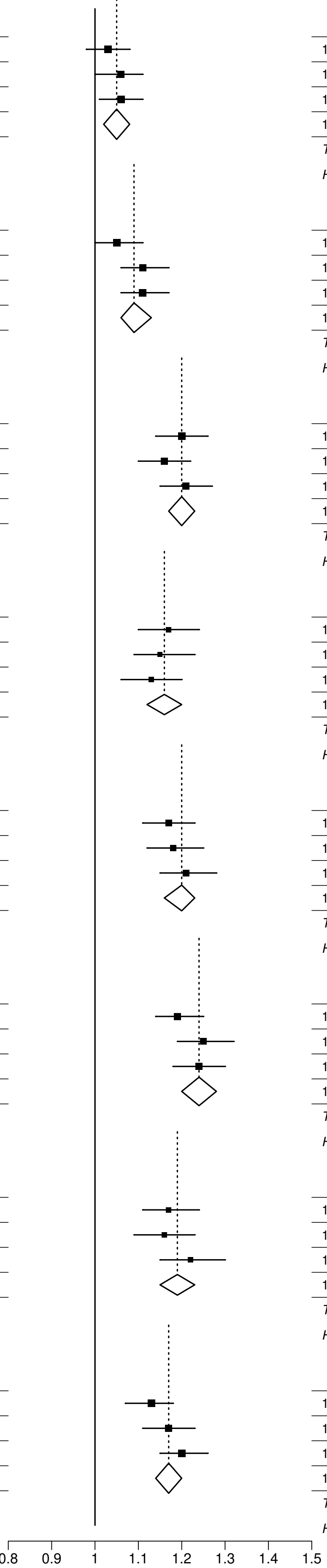

Webfigure 2: Odds of CHD per 1SD increase in each PRS, by baseline age

Subgroup-specific effects are from the fully adjusted model excluding age. The overall effect shown is from the fully adjusted model as shown in Figure 2.

PRS / education level	CHD cases	CHD controls	OR per SD (95% CI)	AUC (95%CI)
**Tada et al/PGS000011/ European/44 SNPs**				
University/high school	564	20,145	1.11 (1.02-1.21)	0.792 (0.774-0.811)
Middle school	958	32,474	1.02 (0.96-1.09)	0.775 (0.760-0.790)
Elementary	2,841	60,134	1.05 (1.01-1.09)	0.730 (0.721-0.738)
Other	800	15,291	1.05 (0.98-1.13)	0.652 (0.634-0.670)
Overall	5,163	128,044	1.05 (1.02-1.08)	0.745 (0.739-0.752)
			Trend: X^2(df=1)=0.29 (p=0.59)	
			Heterogeneity:Q=2.4 (p=0.49)	
**Oni-Orisan et al/PGS004595/ European/141 SNPs**				
University/high school	564	20,145	1.22 (1.12-1.33)	0.794 (0.775-0.812)
Middle school	958	32,474	1.07 (1.00-1.14)	0.776 (0.761-0.790)
Elementary	2,841	60,134	1.06 (1.02-1.10)	0.730 (0.721-0.738)
Other	800	15,291	1.15 (1.07-1.24)	0.654 (0.636-0.673)
Overall	5,163	128,044	1.09 (1.06-1.13)	0.746 (0.739-0.752)
			Trend: X^2(df=1)=1.14 (p=0.29)	
			Heterogeneity:Q=11.12 (p=0.011)	
**Koyama et al/PGS000337/ Japanese and European/64,185 SNPs**				
University/high school	564	20,145	1.29 (1.19-1.41)	0.797 (0.778-0.815)
Middle school	958	32,474	1.22 (1.14-1.30)	0.778 (0.763-0.792)
Elementary	2,841	60,134	1.20 (1.15-1.25)	0.733 (0.724-0.741)
Other	800	15,291	1.12 (1.04-1.21)	0.653 (0.634-0.671)
Overall	5,163	128,044	1.20 (1.17-1.23)	0.748 (0.742-0.754)
			Trend: X^2(df=1)=5.76 (p=0.016)	
			Heterogeneity:Q=6.43 (p=0.092)	
**Tcheandjieu et al/PGS003446/ Multi-ancestry/485,464 SNPs**				
University/high school	564	20,145	1.32 (1.19-1.47)	0.795 (0.777-0.814)
Middle school	958	32,474	1.15 (1.06-1.24)	0.776 (0.761-0.790)
Elementary	2,841	60,134	1.14 (1.09-1.20)	0.730 (0.722-0.739)
Other	800	15,291	1.13 (1.03-1.23)	0.653 (0.634-0.671)
Overall	5,163	128,044	1.16 (1.12-1.20)	0.746 (0.740-0.753)
			Trend: X^2(df=1)=4.28 (p=0.039)	
			Heterogeneity:Q=6.78 (p=0.079)	
**Tamlander et al/ PGS001780/ European/1,087,958 SNPs**				
University/high school	564	20,145	1.31 (1.19-1.43)	0.795 (0.776-0.814)
Middle school	958	32,474	1.19 (1.11-1.28)	0.777 (0.762-0.792)
Elementary	2,841	60,134	1.20 (1.15-1.25)	0.732 (0.723-0.741)
Other	800	15,291	1.10 (1.02-1.19)	0.652 (0.634-0.670)
Overall	5,163	128,044	1.20 (1.16-1.23)	0.748 (0.741-0.754)
			Trend: X^2(df=1)=6.21 (p=0.013)	
			Heterogeneity:Q=8.38 (p=0.039)	
**Patel et al/PGS003725/ Multi-ancestry/1,273,824 SNPs**				
University/high school	564	20,145	1.40 (1.29-1.53)	0.797 (0.778-0.816)
Middle school	958	32,474	1.26 (1.18-1.35)	0.778 (0.763-0.793)
Elementary	2,841	60,134	1.22 (1.17-1.27)	0.733 (0.724-0.742)
Other	800	15,291	1.17 (1.09-1.26)	0.654 (0.635-0.672)
Overall	5,163	128,044	1.24 (1.20-1.28)	0.749 (0.743-0.755)
			Trend: X^2(df=1)=9.96 (p=0.0016)	
			Heterogeneity:Q=10.91 (p=0.012)	
**Inouye et al/PGS000018/ European/1,720,068 SNPs**				
University/high school	564	20,145	1.31 (1.19-1.45)	0.795 (0.776-0.814)
Middle school	958	32,474	1.19 (1.10-1.29)	0.777 (0.762-0.791)
Elementary	2,841	60,134	1.18 (1.13-1.24)	0.731 (0.722-0.740)
Other	800	15,291	1.15 (1.05-1.26)	0.653 (0.635-0.672)
Overall	5,163	128,044	1.19 (1.15-1.23)	0.747 (0.741-0.753)
			Trend: X^2(df=1)=3.36 (p=0.067)	
			Heterogeneity:Q=4.3 (p=0.23)	
**Khera et al/PGS000013/ European/6,472,620 SNPs**				
University/high school	564	20,145	1.25 (1.15-1.36)	0.794 (0.775-0.813)
Middle school	958	32,474	1.17 (1.10-1.25)	0.776 (0.762-0.791)
Elementary	2,841	60,134	1.17 (1.13-1.22)	0.732 (0.723-0.741)
Other	800	15,291	1.11 (1.03-1.19)	0.652 (0.634-0.671)
Overall	5,163	128,044	1.17 (1.14-1.20)	0.747 (0.741-0.754)
			Trend: X^2(df=1)=3.62 (p=0.057)	
			Heterogeneity:Q=4.47 (p=0.21)	

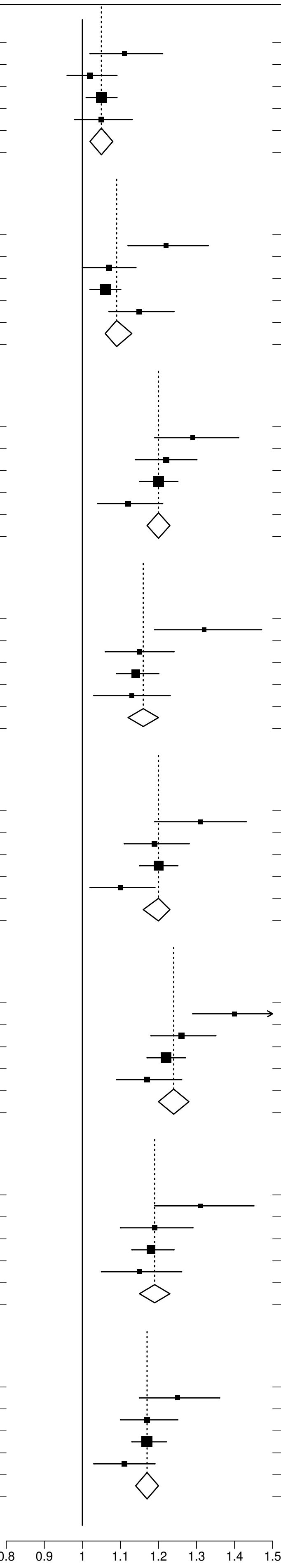

Webfigure 3: Odds of CHD per 1SD increase in each PRS, by highest level of education
Subgroup-specific effects are from the fully adjusted model excluding highest level of education. The overall effect shown is from the fully adjusted model as shown in Figure 2.

PRS / waist-to-hip Ratio	CHD cases	CHD controls	OR per SD (95% CI)	AUC (95%CI)
**Tada et al/PGS000011/ European/44 SNPs**				
<0.85	682	30,630	1.01 (0.93-1.09)	0.775 (0.758-0.792)
0.85 to <0.9	1,171	34,786	1.06 (1.00-1.12)	0.752 (0.738-0.765)
≥0.9	3,310	62,628	1.05 (1.02-1.09)	0.710 (0.702-0.718)
Overall	5,163	128,044	1.05 (1.02-1.08)	0.745 (0.739-0.752)
			Trend: X^2(df=1)=0.41 (p=0.52)	
			Heterogeneity:Q=1.02 (p=0.6)	
**Oni-Orisan et al/PGS004595/ European/141 SNPs**				
<0.85	682	30,630	1.04 (0.96-1.13)	0.775 (0.758-0.792)
0.85 to <0.9	1,171	34,786	1.06 (1.00-1.12)	0.752 (0.738-0.765)
≥0.9	3,310	62,628	1.12 (1.08-1.16)	0.711 (0.702-0.719)
Overall	5,163	128,044	1.09 (1.06-1.13)	0.746 (0.739-0.752)
			Trend: X^2(df=1)=4.16 (p=0.041)	
			Heterogeneity:Q=4.39 (p=0.11)	
**Koyama et al/PGS000337/ Japanese and European/64,185 SNPs**				
<0.85	682	30,630	1.17 (1.08-1.26)	0.776 (0.758-0.793)
0.85 to <0.9	1,171	34,786	1.09 (1.03-1.16)	0.753 (0.739-0.766)
≥0.9	3,310	62,628	1.24 (1.20-1.29)	0.715 (0.707-0.724)
Overall	5,163	128,044	1.20 (1.17-1.23)	0.748 (0.742-0.754)
			Trend: X^2(df=1)=6.2 (p=0.013)	
			Heterogeneity:Q=13.13 (p=0.0014)	
**Tcheandjieu et al/PGS003446/ Multi-ancestry/485,464 SNPs**				
<0.85	682	30,630	1.02 (0.93-1.12)	0.774 (0.757-0.792)
0.85 to <0.9	1,171	34,786	1.08 (1.00-1.16)	0.752 (0.738-0.766)
≥0.9	3,310	62,628	1.22 (1.17-1.27)	0.713 (0.704-0.721)
Overall	5,163	128,044	1.16 (1.12-1.20)	0.746 (0.740-0.753)
			Trend: X^2(df=1)=15.78 (p<0.001)	
			Heterogeneity:Q=16.29 (p<0.001)	
**Tamlander et al/ PGS001780/ European/1,087,958 SNPs**				
<0.85	682	30,630	1.11 (1.03-1.21)	0.775 (0.758-0.793)
0.85 to <0.9	1,171	34,786	1.15 (1.07-1.22)	0.753 (0.739-0.766)
≥0.9	3,310	62,628	1.23 (1.19-1.28)	0.714 (0.706-0.722)
Overall	5,163	128,044	1.20 (1.16-1.23)	0.748 (0.741-0.754)
			Trend: X^2(df=1)=6.49 (p=0.011)	
			Heterogeneity:Q=6.65 (p=0.036)	
**Patel et al/PGS003725/ Multi-ancestry/1,273,824 SNPs**				
<0.85	682	30,630	1.11 (1.03-1.21)	0.776 (0.758-0.793)
0.85 to <0.9	1,171	34,786	1.19 (1.12-1.26)	0.754 (0.740-0.767)
≥0.9	3,310	62,628	1.28 (1.24-1.33)	0.717 (0.708-0.725)
Overall	5,163	128,044	1.24 (1.20-1.28)	0.749 (0.743-0.755)
			Trend: X^2(df=1)=12.19 (p<0.001)	
			Heterogeneity:Q=12.19 (p=0.0023)	
**Inouye et al/PGS000018/ European/1,720,068 SNPs**				
<0.85	682	30,630	1.11 (1.01-1.21)	0.776 (0.759-0.793)
0.85 to <0.9	1,171	34,786	1.11 (1.04-1.20)	0.752 (0.739-0.766)
≥0.9	3,310	62,628	1.24 (1.19-1.30)	0.714 (0.705-0.722)
Overall	5,163	128,044	1.19 (1.15-1.23)	0.747 (0.741-0.753)
			Trend: X^2(df=1)=7.82 (p=0.0052)	
			Heterogeneity:Q=9.33 (p=0.0094)	
**Khera et al/PGS000013/ European/6,472,620 SNPs**				
<0.85	682	30,630	1.09 (1.01-1.18)	0.775 (0.758-0.792)
0.85 to <0.9	1,171	34,786	1.11 (1.05-1.18)	0.753 (0.739-0.766)
≥0.9	3,310	62,628	1.21 (1.17-1.25)	0.714 (0.705-0.722)
Overall	5,163	128,044	1.17 (1.14-1.20)	0.747 (0.741-0.754)
			Trend: X^2(df=1)=8.92 (p=0.0028)	
			Heterogeneity:Q=9.76 (p=0.0076)	

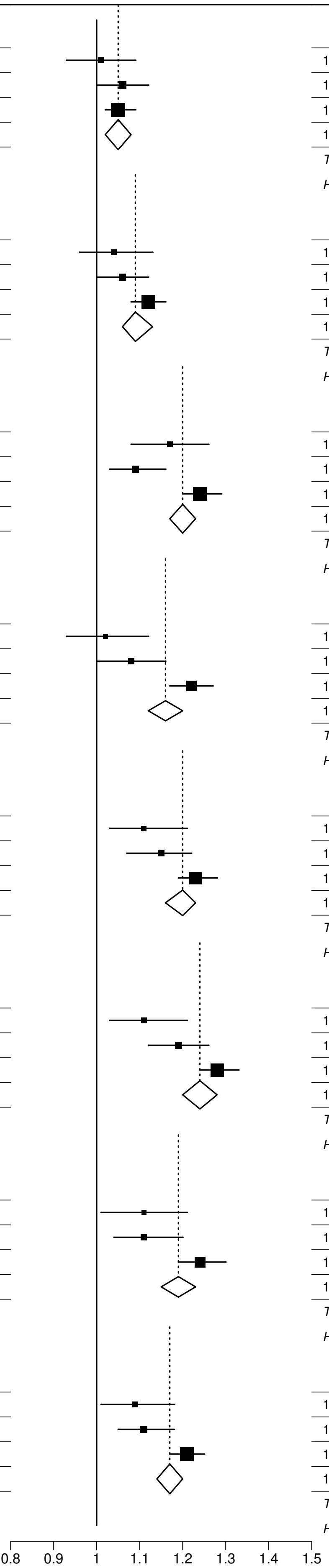

Webfigure 4: Odds of CHD per 1SD increase in each PRS, by waist-to-hip ratio
Subgroup-specific effects are from the fully adjusted model excluding waist-to-hip ratio. The overall effect shown is from the fully adjusted model as shown in Figure 2.

PRS / systolic blood pressure, mmHg	CHD cases	CHD controls	OR per SD (95% CI)	AUC (95%CI)
**Tada et al/PGS000011/ European/44 SNPs**				
<120 mmHg	990	39,975	1.06 (1.00-1.13)	0.773 (0.758-0.789)
120-139 mmHg	2,411	63,641	1.08 (1.04-1.13)	0.734 (0.725-0.744)
≥140 mmHg	1,762	24,428	1.00 (0.95-1.05)	0.656 (0.643-0.668)
Overall	5,163	128,044	1.05 (1.02-1.08)	0.745 (0.739-0.752)
			Trend: X²(df=1)=3.09 (p=0.079)	
			Heterogeneity:Q=5.75 (p=0.056)	
**Oni-Orisan et al/PGS004595/ European/141 SNPs**				
<120 mmHg	990	39,975	1.10 (1.03-1.17)	0.775 (0.760-0.790)
120-139 mmHg	2,411	63,641	1.09 (1.05-1.14)	0.735 (0.725-0.744)
≥140 mmHg	1,762	24,428	1.10 (1.04-1.15)	0.657 (0.644-0.670)
Overall	5,163	128,044	1.09 (1.06-1.13)	0.746 (0.739-0.752)
			Trend: X²(df=1)=0 (p=0.95)	
			Heterogeneity:Q=0.1 (p=0.95)	
**Koyama et al/PGS000337/ Japanese and European/64,185 SNPs**				
<120 mmHg	990	39,975	1.29 (1.21-1.38)	0.779 (0.764-0.794)
120-139 mmHg	2,411	63,641	1.20 (1.15-1.25)	0.737 (0.727-0.746)
≥140 mmHg	1,762	24,428	1.15 (1.10-1.21)	0.659 (0.646-0.672)
Overall	5,163	128,044	1.20 (1.17-1.23)	0.748 (0.742-0.754)
			Trend: X²(df=1)=7.32 (p=0.0068)	
			Heterogeneity:Q=7.56 (p=0.023)	
**Tcheandjieu et al/PGS003446/ Multi-ancestry/485,464 SNPs**				
<120 mmHg	990	39,975	1.19 (1.10-1.28)	0.774 (0.758-0.789)
120-139 mmHg	2,411	63,641	1.18 (1.12-1.24)	0.736 (0.726-0.745)
≥140 mmHg	1,762	24,428	1.11 (1.05-1.18)	0.657 (0.644-0.669)
Overall	5,163	128,044	1.16 (1.12-1.20)	0.746 (0.740-0.753)
			Trend: X²(df=1)=2.41 (p=0.12)	
			Heterogeneity:Q=2.95 (p=0.23)	
**Tamlander et al/ PGS001780/ European/1,087,958 SNPs**				
<120 mmHg	990	39,975	1.21 (1.12-1.29)	0.774 (0.759-0.789)
120-139 mmHg	2,411	63,641	1.23 (1.17-1.28)	0.737 (0.727-0.746)
≥140 mmHg	1,762	24,428	1.15 (1.09-1.21)	0.659 (0.646-0.672)
Overall	5,163	128,044	1.20 (1.16-1.23)	0.748 (0.741-0.754)
			Trend: X²(df=1)=2 (p=0.16)	
			Heterogeneity:Q=3.73 (p=0.15)	
**Patel et al/PGS003725/ Multi-ancestry/1,273,824 SNPs**				
<120 mmHg	990	39,975	1.29 (1.21-1.38)	0.778 (0.763-0.793)
120-139 mmHg	2,411	63,641	1.26 (1.21-1.32)	0.738 (0.728-0.747)
≥140 mmHg	1,762	24,428	1.18 (1.12-1.24)	0.660 (0.648-0.673)
Overall	5,163	128,044	1.24 (1.20-1.28)	0.749 (0.743-0.755)
			Trend: X²(df=1)=5.2 (p=0.023)	
			Heterogeneity:Q=5.68 (p=0.058)	
**Inouye et al/PGS000018/ European/1,720,068 SNPs**				
<120 mmHg	990	39,975	1.23 (1.14-1.33)	0.775 (0.759-0.790)
120-139 mmHg	2,411	63,641	1.23 (1.17-1.29)	0.736 (0.727-0.746)
≥140 mmHg	1,762	24,428	1.12 (1.06-1.19)	0.657 (0.645-0.670)
Overall	5,163	128,044	1.19 (1.15-1.23)	0.747 (0.741-0.753)
			Trend: X²(df=1)=4.75 (p=0.029)	
			Heterogeneity:Q=6.49 (p=0.039)	
**Khera et al/PGS000013/ European/6,472,620 SNPs**				
<120 mmHg	990	39,975	1.18 (1.11-1.26)	0.774 (0.759-0.790)
120-139 mmHg	2,411	63,641	1.20 (1.15-1.25)	0.736 (0.727-0.746)
≥140 mmHg	1,762	24,428	1.13 (1.08-1.19)	0.659 (0.646-0.672)
Overall	5,163	128,044	1.17 (1.14-1.20)	0.747 (0.741-0.754)
			Trend: X²(df=1)=1.76 (p=0.18)	
			Heterogeneity:Q=3.47 (p=0.18)	

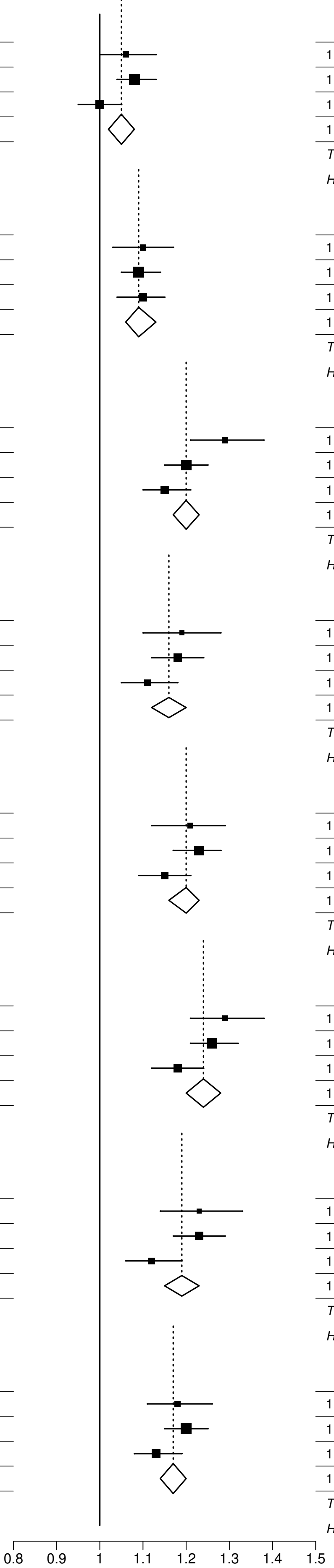

Webfigure 5: Odds of CHD per 1SD increase in each PRS, by systolic blood pressure

Subgroup-specific effects are from the fully adjusted model excluding systolic blood pressure. The overall effect shown is from the fully adjusted model as shown in Figure 2.

PRS / diastolic blood pressure, mmHg	CHD cases	CHD controls	OR per SD (95% CI)	AUC (95%CI)
**Tada et al/PGS000011/ European/44 SNPs**				
<80 mmHg	1,246	40,134	1.07 (1.01-1.13)	0.774 (0.761-0.787)
80-89 mmHg	2,028	55,371	1.05 (1.01-1.10)	0.747 (0.737-0.757)
≥90 mmHg	1,889	32,539	1.03 (0.99-1.08)	0.689 (0.677-0.700)
Overall	5,163	128,044	1.05 (1.02-1.08)	0.745 (0.739-0.752)
			Trend: X²(df=1)=1.03 (p=0.31)	
			Heterogeneity:Q=1.03 (p=0.6)	
**Oni-Orisan et al/PGS004595/ European/141 SNPs**				
<80 mmHg	1,246	40,134	1.10 (1.04-1.17)	0.774 (0.761-0.787)
80-89 mmHg	2,028	55,371	1.09 (1.04-1.14)	0.748 (0.738-0.758)
≥90 mmHg	1,889	32,539	1.09 (1.04-1.14)	0.689 (0.678-0.701)
Overall	5,163	128,044	1.09 (1.06-1.13)	0.746 (0.739-0.752)
			Trend: X²(df=1)=0.05 (p=0.82)	
			Heterogeneity:Q=0.07 (p=0.96)	
**Koyama et al/PGS000337/ Japanese and European/64,185 SNPs**				
<80 mmHg	1,246	40,134	1.28 (1.21-1.36)	0.779 (0.765-0.792)
80-89 mmHg	2,028	55,371	1.19 (1.13-1.24)	0.749 (0.739-0.760)
≥90 mmHg	1,889	32,539	1.16 (1.10-1.21)	0.691 (0.679-0.702)
Overall	5,163	128,044	1.20 (1.17-1.23)	0.748 (0.742-0.754)
			Trend: X²(df=1)=6.15 (p=0.013)	
			Heterogeneity:Q=6.76 (p=0.034)	
**Tcheandjieu et al/PGS003446/ Multi-ancestry/485,464 SNPs**				
<80 mmHg	1,246	40,134	1.19 (1.11-1.27)	0.774 (0.761-0.788)
80-89 mmHg	2,028	55,371	1.15 (1.09-1.21)	0.748 (0.738-0.758)
≥90 mmHg	1,889	32,539	1.14 (1.08-1.21)	0.690 (0.678-0.701)
Overall	5,163	128,044	1.16 (1.12-1.20)	0.746 (0.740-0.753)
			Trend: X²(df=1)=0.8 (p=0.37)	
			Heterogeneity:Q=0.92 (p=0.63)	
**Tamlander et al/ PGS001780/ European/1,087,958 SNPs**				
<80 mmHg	1,246	40,134	1.24 (1.17-1.32)	0.775 (0.762-0.788)
80-89 mmHg	2,028	55,371	1.19 (1.13-1.25)	0.749 (0.739-0.759)
≥90 mmHg	1,889	32,539	1.17 (1.11-1.23)	0.692 (0.680-0.703)
Overall	5,163	128,044	1.20 (1.16-1.23)	0.748 (0.741-0.754)
			Trend: X²(df=1)=1.91 (p=0.17)	
			Heterogeneity:Q=2.05 (p=0.36)	
**Patel et al/PGS003725/ Multi-ancestry/1,273,824 SNPs**				
<80 mmHg	1,246	40,134	1.30 (1.22-1.37)	0.779 (0.766-0.792)
80-89 mmHg	2,028	55,371	1.24 (1.18-1.29)	0.750 (0.740-0.760)
≥90 mmHg	1,889	32,539	1.20 (1.14-1.26)	0.692 (0.680-0.704)
Overall	5,163	128,044	1.24 (1.20-1.28)	0.749 (0.743-0.755)
			Trend: X²(df=1)=4.19 (p=0.041)	
			Heterogeneity:Q=4.24 (p=0.12)	
**Inouye et al/PGS000018/ European/1,720,068 SNPs**				
<80 mmHg	1,246	40,134	1.24 (1.16-1.33)	0.775 (0.762-0.788)
80-89 mmHg	2,028	55,371	1.22 (1.15-1.28)	0.749 (0.739-0.759)
≥90 mmHg	1,889	32,539	1.13 (1.07-1.20)	0.691 (0.679-0.702)
Overall	5,163	128,044	1.19 (1.15-1.23)	0.747 (0.741-0.753)
			Trend: X²(df=1)=4.66 (p=0.031)	
			Heterogeneity:Q=5.37 (p=0.068)	
**Khera et al/PGS000013/ European/6,472,620 SNPs**				
<80 mmHg	1,246	40,134	1.18 (1.12-1.25)	0.775 (0.762-0.788)
80-89 mmHg	2,028	55,371	1.17 (1.12-1.22)	0.749 (0.739-0.759)
≥90 mmHg	1,889	32,539	1.16 (1.11-1.22)	0.692 (0.681-0.704)
Overall	5,163	128,044	1.17 (1.14-1.20)	0.747 (0.741-0.754)
			Trend: X²(df=1)=0.21 (p=0.65)	
			Heterogeneity:Q=0.21 (p=0.9)	

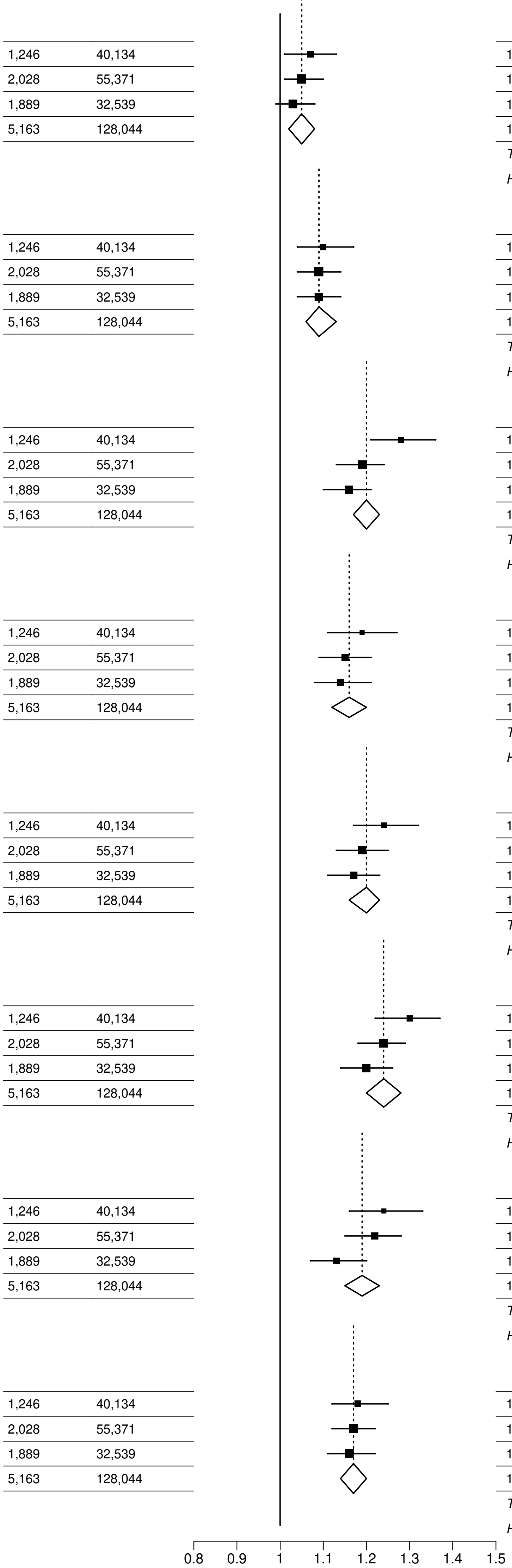

Webfigure 6: Odds of CHD per 1SD increase in each PRS, by diastolic blood pressure
Subgroup-specific effects are from the fully adjusted model excluding diastolic blood pressure. The overall effect shown is from the fully adjusted model as shown in Figure 2.

PRS / smoking status group	CHD cases	CHD controls	OR per SD (95% CI)	AUC (95%CI)
**Tada et al/PGS000011/ European/44 SNPs**				
Never	2,166	62,535	1.07 (1.02-1.12)	0.738 (0.728-0.747)
Former smoker	1,467	24,709	1.04 (0.98-1.09)	0.724 (0.712-0.736)
Current smoker	1,530	40,800	1.03 (0.98-1.08)	0.756 (0.745-0.768)
Overall	5,163	128,044	1.05 (1.02-1.08)	0.745 (0.739-0.752)
			Trend: $X^2(df=1)=1.3$ (p=0.25)	
			Heterogeneity:Q=1.38 (p=0.5)	
**Oni-Orisan et al/PGS004595/ European/141 SNPs**				
Never	2,166	62,535	1.06 (1.02-1.11)	0.737 (0.727-0.747)
Former smoker	1,467	24,709	1.15 (1.09-1.21)	0.726 (0.713-0.738)
Current smoker	1,530	40,800	1.09 (1.03-1.15)	0.757 (0.745-0.768)
Overall	5,163	128,044	1.09 (1.06-1.13)	0.746 (0.739-0.752)
			Trend: $X^2(df=1)=0.98$ (p=0.32)	
			Heterogeneity:Q=5.29 (p=0.071)	
**Koyama et al/PGS000337/ Japanese and European/64,185 SNPs**				
Never	2,166	62,535	1.14 (1.09-1.19)	0.738 (0.728-0.748)
Former smoker	1,467	24,709	1.27 (1.21-1.35)	0.730 (0.718-0.742)
Current smoker	1,530	40,800	1.22 (1.16-1.29)	0.762 (0.750-0.773)
Overall	5,163	128,044	1.20 (1.17-1.23)	0.748 (0.742-0.754)
			Trend: $X^2(df=1)=4.61$ (p=0.032)	
			Heterogeneity:Q=9.68 (p=0.0079)	
**Tcheandjieu et al/PGS003446/ Multi-ancestry/485,464 SNPs**				
Never	2,166	62,535	1.05 (0.99-1.10)	0.737 (0.727-0.747)
Former smoker	1,467	24,709	1.29 (1.21-1.38)	0.728 (0.716-0.741)
Current smoker	1,530	40,800	1.22 (1.14-1.30)	0.760 (0.748-0.771)
Overall	5,163	128,044	1.16 (1.12-1.20)	0.746 (0.740-0.753)
			Trend: $X^2(df=1)=15.19$ (p<0.001)	
			Heterogeneity:Q=26.1 (p<0.001)	
**Tamlander et al/ PGS001780/ European/1,087,958 SNPs**				
Never	2,166	62,535	1.13 (1.08-1.19)	0.738 (0.728-0.748)
Former smoker	1,467	24,709	1.25 (1.18-1.32)	0.729 (0.716-0.741)
Current smoker	1,530	40,800	1.25 (1.18-1.32)	0.761 (0.750-0.773)
Overall	5,163	128,044	1.20 (1.16-1.23)	0.748 (0.741-0.754)
			Trend: $X^2(df=1)=8.14$ (p=0.0043)	
			Heterogeneity:Q=10.22 (p=0.006)	
**Patel et al/PGS003725/ Multi-ancestry/1,273,824 SNPs**				
Never	2,166	62,535	1.17 (1.12-1.23)	0.738 (0.729-0.748)
Former smoker	1,467	24,709	1.31 (1.24-1.38)	0.731 (0.719-0.744)
Current smoker	1,530	40,800	1.28 (1.21-1.35)	0.763 (0.752-0.774)
Overall	5,163	128,044	1.24 (1.20-1.28)	0.749 (0.743-0.755)
			Trend: $X^2(df=1)=7.48$ (p=0.0062)	
			Heterogeneity:Q=11.66 (p=0.0029)	
**Inouye et al/PGS000018/ European/1,720,068 SNPs**				
Never	2,166	62,535	1.13 (1.07-1.19)	0.738 (0.728-0.748)
Former smoker	1,467	24,709	1.28 (1.20-1.36)	0.729 (0.716-0.741)
Current smoker	1,530	40,800	1.21 (1.14-1.29)	0.759 (0.748-0.771)
Overall	5,163	128,044	1.19 (1.15-1.23)	0.747 (0.741-0.753)
			Trend: $X^2(df=1)=3.44$ (p=0.064)	
			Heterogeneity:Q=8.82 (p=0.012)	
**Khera et al/PGS000013/ European/6,472,620 SNPs**				
Never	2,166	62,535	1.13 (1.08-1.18)	0.738 (0.728-0.748)
Former smoker	1,467	24,709	1.19 (1.13-1.26)	0.727 (0.715-0.739)
Current smoker	1,530	40,800	1.21 (1.15-1.27)	0.761 (0.749-0.772)
Overall	5,163	128,044	1.17 (1.14-1.20)	0.747 (0.741-0.754)
			Trend: $X^2(df=1)=4.14$ (p=0.042)	
			Heterogeneity:Q=4.44 (p=0.11)	

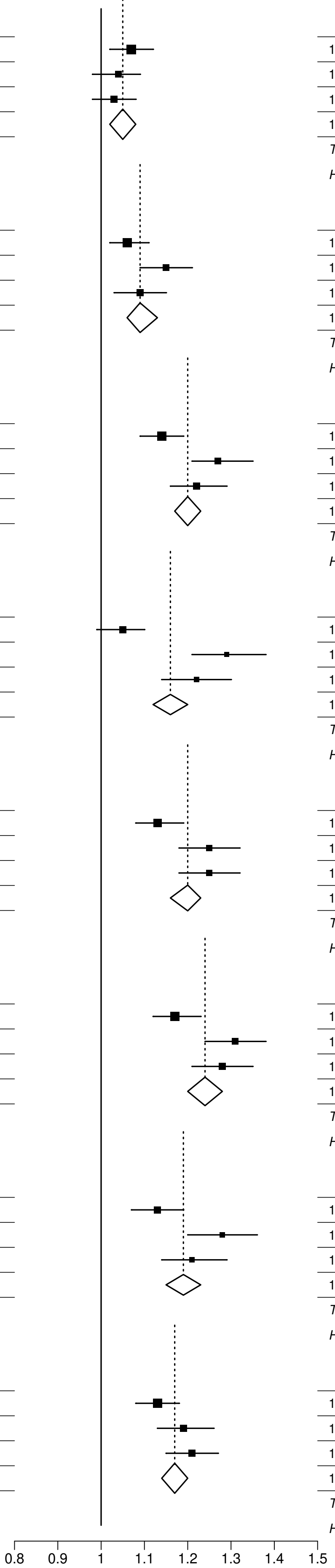

Webfigure 7: Odds of CHD per 1SD increase in each PRS, by smoking status
Subgroup-specific effects are from the fully adjusted model excluding smoking status. The overall effect shown is from the fully adjusted model as shown in Figure 2.

PRS / diabetes at baseline	CHD cases	CHD controls	OR per SD (95% CI)	AUC (95%CI)
**Tada et al/PGS000011/ European/44 SNPs**				
No Diabetes	3,064	105,347	1.06 (1.02-1.10)	0.733 (0.725-0.741)
Diabetes	2,099	22,697	1.03 (0.98-1.07)	0.639 (0.627-0.651)
Overall	5,163	128,044	1.05 (1.02-1.08)	0.745 (0.739-0.752)
			Heterogeneity:Q=0.96 (p=0.33)	
**Oni-Orisan et al/PGS004595/ European/141 SNPs**				
No Diabetes	3,064	105,347	1.08 (1.04-1.12)	0.733 (0.725-0.741)
Diabetes	2,099	22,697	1.11 (1.06-1.16)	0.641 (0.629-0.653)
Overall	5,163	128,044	1.09 (1.06-1.13)	0.746 (0.739-0.752)
			Heterogeneity:Q=0.84 (p=0.36)	
**Koyama et al/PGS000337/ Japanese and European/64,185 SNPs**				
No Diabetes	3,064	105,347	1.18 (1.14-1.23)	0.735 (0.727-0.744)
Diabetes	2,099	22,697	1.22 (1.16-1.28)	0.646 (0.634-0.658)
Overall	5,163	128,044	1.20 (1.17-1.23)	0.748 (0.742-0.754)
			Heterogeneity:Q=1.23 (p=0.27)	
**Tcheandjieu et al/PGS003446/ Multi-ancestry/485,464 SNPs**				
No Diabetes	3,064	105,347	1.14 (1.09-1.19)	0.734 (0.725-0.742)
Diabetes	2,099	22,697	1.18 (1.12-1.25)	0.643 (0.631-0.655)
Overall	5,163	128,044	1.16 (1.12-1.20)	0.746 (0.740-0.753)
			Heterogeneity:Q=0.92 (p=0.34)	
**Tamlander et al/ PGS001780/ European/1,087,958 SNPs**				
No Diabetes	3,064	105,347	1.18 (1.14-1.23)	0.735 (0.726-0.743)
Diabetes	2,099	22,697	1.21 (1.15-1.27)	0.646 (0.634-0.658)
Overall	5,163	128,044	1.20 (1.16-1.23)	0.748 (0.741-0.754)
			Heterogeneity:Q=0.62 (p=0.43)	
**Patel et al/PGS003725/ Multi-ancestry/1,273,824 SNPs**				
No Diabetes	3,064	105,347	1.22 (1.17-1.26)	0.736 (0.728-0.744)
Diabetes	2,099	22,697	1.26 (1.21-1.32)	0.651 (0.639-0.663)
Overall	5,163	128,044	1.24 (1.20-1.28)	0.749 (0.743-0.755)
			Heterogeneity:Q=1.13 (p=0.29)	
**Inouye et al/PGS000018/ European/1,720,068 SNPs**				
No Diabetes	3,064	105,347	1.19 (1.14-1.24)	0.734 (0.726-0.743)
Diabetes	2,099	22,697	1.19 (1.13-1.26)	0.643 (0.631-0.655)
Overall	5,163	128,044	1.19 (1.15-1.23)	0.747 (0.741-0.753)
			Heterogeneity:Q=0 (p=1)	
**Khera et al/PGS000013/ European/6,472,620 SNPs**				
No Diabetes	3,064	105,347	1.17 (1.13-1.21)	0.734 (0.726-0.743)
Diabetes	2,099	22,697	1.17 (1.12-1.22)	0.644 (0.632-0.657)
Overall	5,163	128,044	1.17 (1.14-1.20)	0.747 (0.741-0.754)
			Heterogeneity:Q=0 (p=1)	

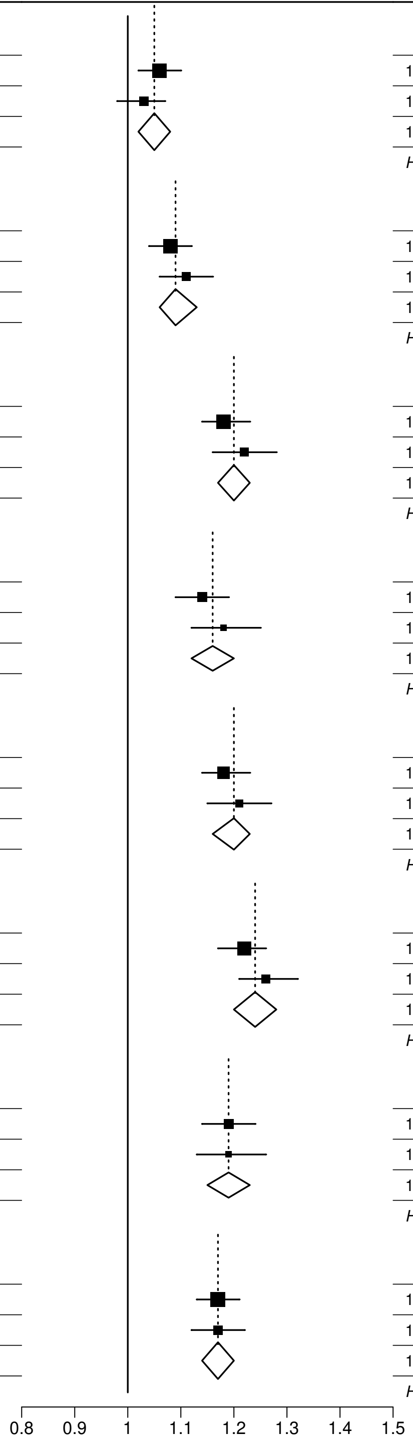

Webfigure 8: Odds of CHD per 1SD increase in each PRS, by baseline diabetes status
Subgroup-specific effects are from the fully adjusted model excluding baseline diabetes status. The overall effect shown is from the fully adjusted model as shown in Figure 2.

PRS / indigenous ancestry proportion	CHD cases	CHD controls	OR per SD (95% CI)	AUC (95%CI)
**Tada et al/PGS000011/ European/44 SNPs**				
<60%	2,131	46,104	1.03 (0.98-1.07)	0.752 (0.742-0.762)
60% to <80%	1,786	46,115	1.08 (1.03-1.13)	0.753 (0.742-0.763)
≥80%	1,159	33,742	1.03 (0.97-1.10)	0.724 (0.710-0.737)
Overall	5,163	128,044	1.05 (1.02-1.08)	0.745 (0.739-0.752)
			Trend: X²(df=1)=0.08 (p=0.78)	
			Heterogeneity:Q=2.45 (p=0.29)	
**Oni-Orisan et al/PGS004595/ European/141 SNPs**				
<60%	2,131	46,104	1.10 (1.06-1.15)	0.753 (0.743-0.762)
60% to <80%	1,786	46,115	1.10 (1.05-1.15)	0.752 (0.742-0.763)
≥80%	1,159	33,742	1.07 (1.00-1.14)	0.724 (0.710-0.737)
Overall	5,163	128,044	1.09 (1.06-1.13)	0.746 (0.739-0.752)
			Trend: X²(df=1)=0.38 (p=0.54)	
			Heterogeneity:Q=0.58 (p=0.75)	
**Koyama et al/PGS000337/ Japanese and European/64,185 SNPs**				
<60%	2,131	46,104	1.19 (1.14-1.24)	0.754 (0.745-0.764)
60% to <80%	1,786	46,115	1.23 (1.17-1.30)	0.756 (0.745-0.766)
≥80%	1,159	33,742	1.17 (1.10-1.25)	0.726 (0.712-0.739)
Overall	5,163	128,044	1.20 (1.17-1.23)	0.748 (0.742-0.754)
			Trend: X²(df=1)=0.03 (p=0.87)	
			Heterogeneity:Q=1.74 (p=0.42)	
**Tcheandjieu et al/PGS003446/ Multi-ancestry/485,464 SNPs**				
<60%	2,131	46,104	1.18 (1.12-1.24)	0.754 (0.744-0.763)
60% to <80%	1,786	46,115	1.16 (1.09-1.23)	0.753 (0.743-0.764)
≥80%	1,159	33,742	1.13 (1.05-1.23)	0.724 (0.710-0.738)
Overall	5,163	128,044	1.16 (1.12-1.20)	0.746 (0.740-0.753)
			Trend: X²(df=1)=0.81 (p=0.37)	
			Heterogeneity:Q=0.82 (p=0.66)	
**Tamlander et al/ PGS001780/ European/1,087,958 SNPs**				
<60%	2,131	46,104	1.19 (1.14-1.25)	0.754 (0.744-0.764)
60% to <80%	1,786	46,115	1.24 (1.18-1.30)	0.756 (0.745-0.766)
≥80%	1,159	33,742	1.15 (1.07-1.23)	0.725 (0.711-0.738)
Overall	5,163	128,044	1.20 (1.16-1.23)	0.748 (0.741-0.754)
			Trend: X²(df=1)=0.19 (p=0.66)	
			Heterogeneity:Q=3.16 (p=0.21)	
**Patel et al/PGS003725/ Multi-ancestry/1,273,824 SNPs**				
<60%	2,131	46,104	1.22 (1.17-1.27)	0.755 (0.746-0.765)
60% to <80%	1,786	46,115	1.30 (1.24-1.37)	0.757 (0.746-0.768)
≥80%	1,159	33,742	1.19 (1.12-1.27)	0.726 (0.712-0.739)
Overall	5,163	128,044	1.24 (1.20-1.28)	0.749 (0.743-0.755)
			Trend: X²(df=1)=0.01 (p=0.93)	
			Heterogeneity:Q=5.61 (p=0.061)	
**Inouye et al/PGS000018/ European/1,720,068 SNPs**				
<60%	2,131	46,104	1.16 (1.11-1.22)	0.753 (0.744-0.763)
60% to <80%	1,786	46,115	1.24 (1.17-1.31)	0.755 (0.744-0.765)
≥80%	1,159	33,742	1.18 (1.09-1.28)	0.725 (0.711-0.739)
Overall	5,163	128,044	1.19 (1.15-1.23)	0.747 (0.741-0.753)
			Trend: X²(df=1)=0.6 (p=0.44)	
			Heterogeneity:Q=2.9 (p=0.23)	
**Khera et al/PGS000013/ European/6,472,620 SNPs**				
<60%	2,131	46,104	1.15 (1.10-1.20)	0.753 (0.744-0.763)
60% to <80%	1,786	46,115	1.24 (1.18-1.30)	0.756 (0.745-0.767)
≥80%	1,159	33,742	1.12 (1.05-1.20)	0.724 (0.710-0.738)
Overall	5,163	128,044	1.17 (1.14-1.20)	0.747 (0.741-0.754)
			Trend: X²(df=1)=0 (p=0.97)	
			Heterogeneity:Q=7.86 (p=0.02)	

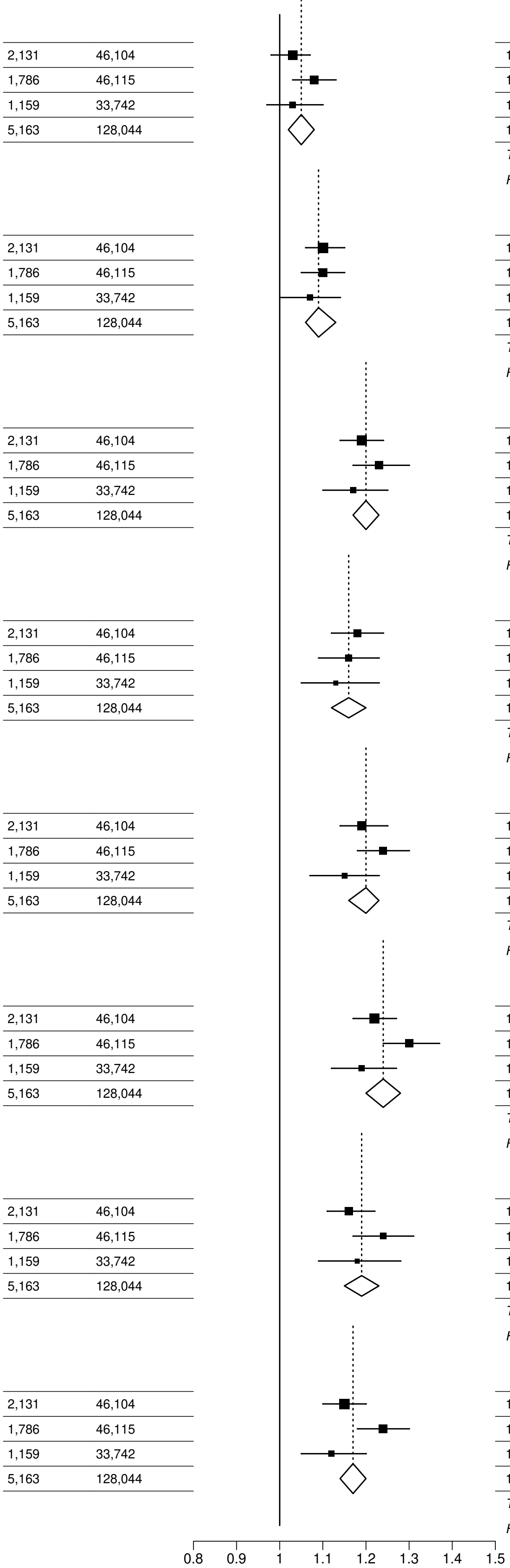

Webfigure 9: Odds of CHD per 1SD increase in each PRS, by level of Indigenous American ancestry

Subgroup-specific effects are from the fully adjusted model.The overall effect shown is from the fully adjusted model as shown in Figure 2.

PRS name	Cases	Controls	OR per SD (95% CI)	AUC (95%CI)
**Tada et al/PGS000011/ European/44 SNPs**				
Partially adjusted	494	132,713	1.07 (0.98−1.17)	0.741 (0.720−0.762)
Fully adjusted	494	132,713	1.07 (0.98−1.17)	0.748 (0.727−0.769)
**Oni−Orisan et al/PGS004595/ European/141 SNPs**				
Partially adjusted	494	132,713	1.05 (0.96−1.15)	0.740 (0.719−0.761)
Fully adjusted	494	132,713	1.05 (0.96−1.15)	0.747 (0.726−0.769)
**Koyama et al/PGS000337/ Japanese and European/64,185 SNPs**				
Partially adjusted	494	132,713	1.21 (1.11−1.33)	0.744 (0.722−0.765)
Fully adjusted	494	132,713	1.21 (1.10−1.32)	0.750 (0.729−0.772)
**Tcheandjieu et al/PGS003446/ Multi−ancestry/485,464 SNPs**				
Partially adjusted	494	132,713	1.11 (1.00−1.24)	0.741 (0.719−0.762)
Fully adjusted	494	132,713	1.11 (1.00−1.23)	0.748 (0.726−0.769)
**Tamlander et al/ PGS001780/ European/1,087,958 SNPs**				
Partially adjusted	494	132,713	1.11 (1.01−1.22)	0.741 (0.720−0.762)
Fully adjusted	494	132,713	1.10 (1.00−1.21)	0.748 (0.727−0.770)
**Patel et al/PGS003725/ Multi−ancestry/1,273,824 SNPs**				
Partially adjusted	494	132,713	1.15 (1.05−1.25)	0.742 (0.721−0.763)
Fully adjusted	494	132,713	1.14 (1.04−1.24)	0.749 (0.727−0.770)
**Inouye et al/PGS000018/ European/1,720,068 SNPs**				
Partially adjusted	494	132,713	1.15 (1.04−1.28)	0.742 (0.721−0.763)
Fully adjusted	494	132,713	1.14 (1.03−1.27)	0.749 (0.727−0.770)
**Khera et al/PGS000013/ European/6,472,620 SNPs**				
Partially adjusted	494	132,713	1.16 (1.06−1.27)	0.744 (0.723−0.765)
Fully adjusted	494	132,713	1.16 (1.06−1.26)	0.751 (0.729−0.772)

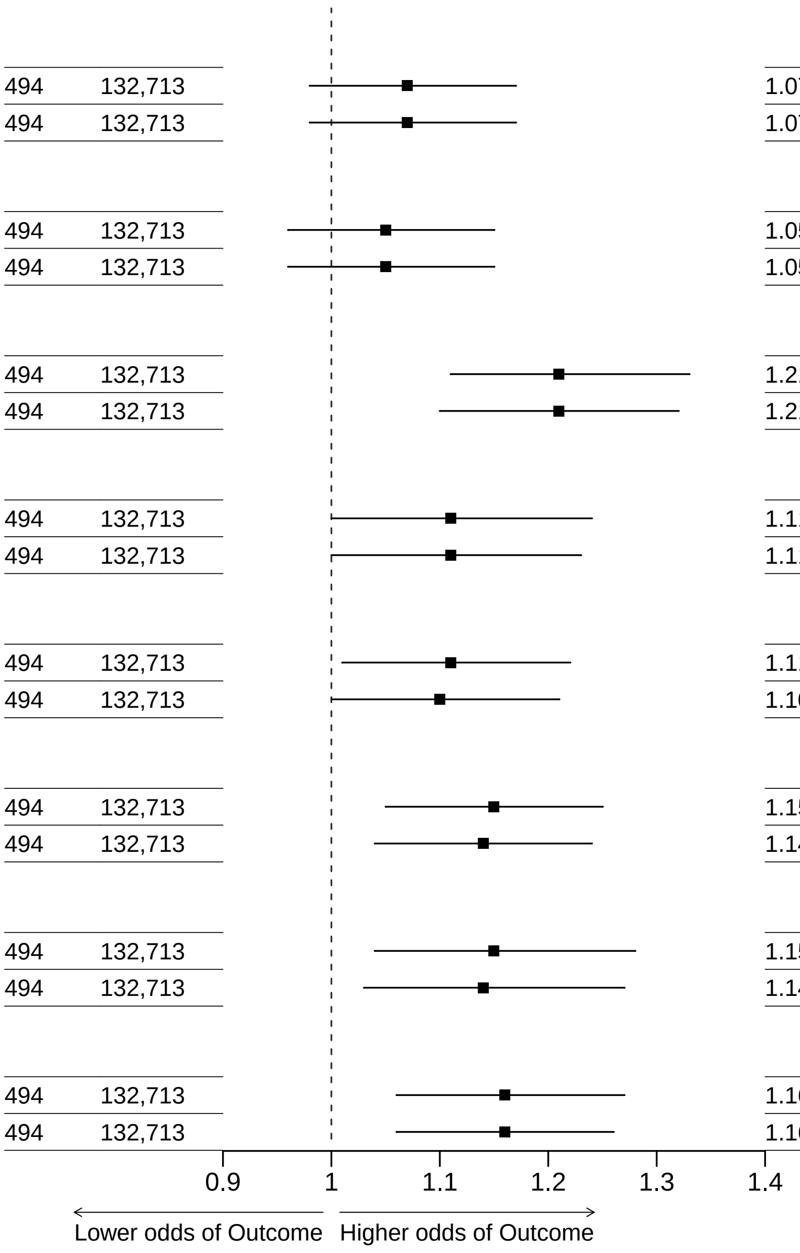

Webfigure 10: Odds of baseline self-reported angina per 1SD increase in each PRS, for participants aged 35-79 years at recruitment
Analyses as for Figure 2.

PRS name	Cases	Controls	OR per SD (95% CI)	AUC (95%CI)
**Tada et al/PGS000011/ European/44 SNPs**				
Partially adjusted	1,476	131,731	1.05 (1.00−1.11)	0.758 (0.745−0.770)
Fully adjusted	1,476	131,731	1.05 (0.99−1.10)	0.771 (0.759−0.783)
**Oni−Orisan et al/PGS004595/ European/141 SNPs**				
Partially adjusted	1,476	131,731	1.13 (1.08−1.19)	0.759 (0.746−0.771)
Fully adjusted	1,476	131,731	1.13 (1.07−1.19)	0.771 (0.759−0.783)
**Koyama et al/PGS000337/ Japanese and European/64,185 SNPs**				
Partially adjusted	1,476	131,731	1.30 (1.24−1.37)	0.764 (0.751−0.776)
Fully adjusted	1,476	131,731	1.29 (1.22−1.36)	0.775 (0.763−0.788)
**Tcheandjieu et al/PGS003446/ Multi−ancestry/485,464 SNPs**				
Partially adjusted	1,476	131,731	1.30 (1.22−1.38)	0.762 (0.750−0.775)
Fully adjusted	1,476	131,731	1.28 (1.20−1.37)	0.774 (0.762−0.786)
**Tamlander et al/ PGS001780/ European/1,087,958 SNPs**				
Partially adjusted	1,476	131,731	1.30 (1.23−1.37)	0.762 (0.750−0.775)
Fully adjusted	1,476	131,731	1.29 (1.22−1.36)	0.774 (0.762−0.786)
**Patel et al/PGS003725/ Multi−ancestry/1,273,824 SNPs**				
Partially adjusted	1,476	131,731	1.36 (1.30−1.44)	0.765 (0.752−0.778)
Fully adjusted	1,476	131,731	1.34 (1.27−1.41)	0.776 (0.763−0.788)
**Inouye et al/PGS000018/ European/1,720,068 SNPs**				
Partially adjusted	1,476	131,731	1.31 (1.23−1.39)	0.762 (0.749−0.774)
Fully adjusted	1,476	131,731	1.30 (1.22−1.38)	0.774 (0.762−0.786)
**Khera et al/PGS000013/ European/6,472,620 SNPs**				
Partially adjusted	1,476	131,731	1.26 (1.19−1.32)	0.762 (0.749−0.774)
Fully adjusted	1,476	131,731	1.25 (1.19−1.31)	0.774 (0.762−0.786)

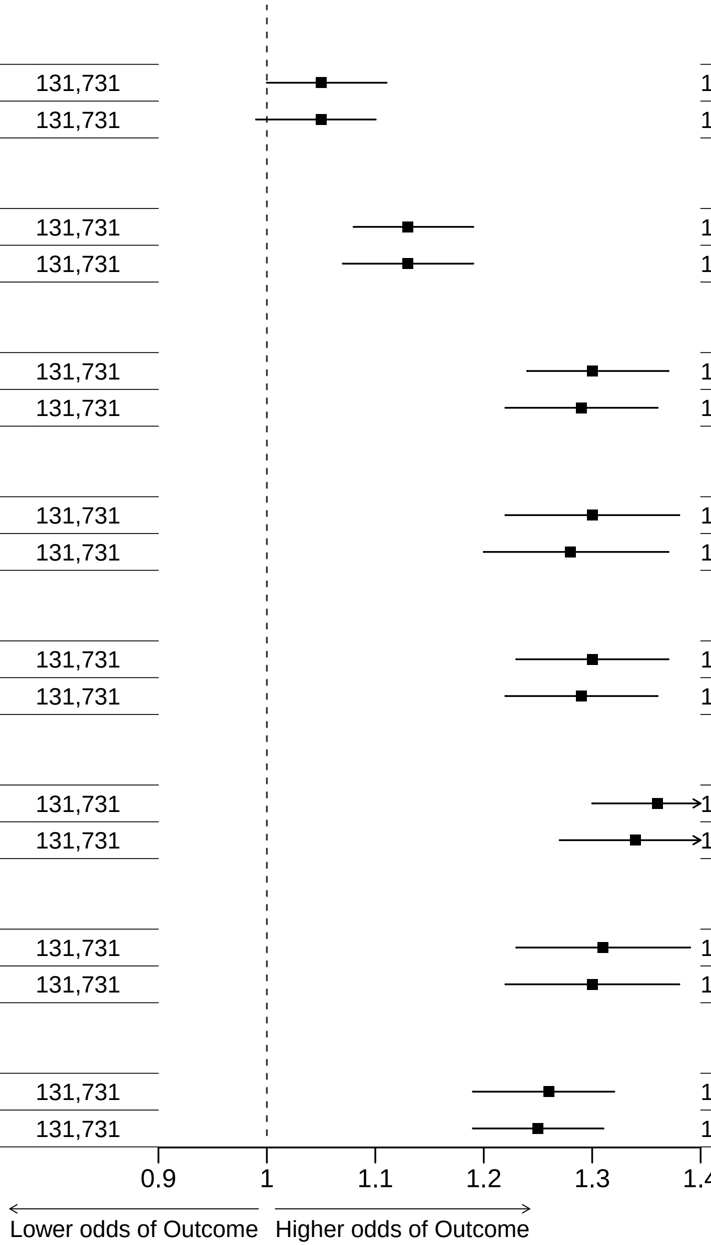

Webfigure 11: Odds of baseline self-reported myocardial infarction per 1SD increase in each PRS, for participants aged 35-79 years at recruitment
Analyses as for Figure 2.

PRS name	Cases	Controls	OR per SD (95% CI)	AUC (95%CI)
**Tada et al/PGS000011/ European/44 SNPs**				
Partially adjusted	1,901	131,306	1.05 (1.01−1.10)	0.748 (0.738−0.759)
Fully adjusted	1,901	131,306	1.05 (1.00−1.10)	0.760 (0.750−0.771)
**Oni−Orisan et al/PGS004595/ European/141 SNPs**				
Partially adjusted	1,901	131,306	1.11 (1.06−1.16)	0.749 (0.738−0.760)
Fully adjusted	1,901	131,306	1.11 (1.06−1.16)	0.761 (0.750−0.771)
**Koyama et al/PGS000337/ Japanese and European/64,185 SNPs**				
Partially adjusted	1,901	131,306	1.28 (1.22−1.34)	0.754 (0.743−0.765)
Fully adjusted	1,901	131,306	1.27 (1.21−1.32)	0.764 (0.753−0.775)
**Tcheandjieu et al/PGS003446/ Multi−ancestry/485,464 SNPs**				
Partially adjusted	1,901	131,306	1.25 (1.18−1.32)	0.751 (0.741−0.762)
Fully adjusted	1,901	131,306	1.23 (1.16−1.30)	0.763 (0.752−0.773)
**Tamlander et al/ PGS001780/ European/1,087,958 SNPs**				
Partially adjusted	1,901	131,306	1.24 (1.18−1.31)	0.752 (0.741−0.763)
Fully adjusted	1,901	131,306	1.23 (1.17−1.29)	0.763 (0.752−0.774)
**Patel et al/PGS003725/ Multi−ancestry/1,273,824 SNPs**				
Partially adjusted	1,901	131,306	1.30 (1.24−1.36)	0.753 (0.742−0.765)
Fully adjusted	1,901	131,306	1.27 (1.22−1.34)	0.763 (0.753−0.774)
**Inouye et al/PGS000018/ European/1,720,068 SNPs**				
Partially adjusted	1,901	131,306	1.26 (1.20−1.33)	0.752 (0.741−0.763)
Fully adjusted	1,901	131,306	1.25 (1.18−1.32)	0.763 (0.752−0.774)
**Khera et al/PGS000013/ European/6,472,620 SNPs**				
Partially adjusted	1,901	131,306	1.23 (1.17−1.28)	0.752 (0.741−0.763)
Fully adjusted	1,901	131,306	1.22 (1.17−1.28)	0.763 (0.752−0.774)

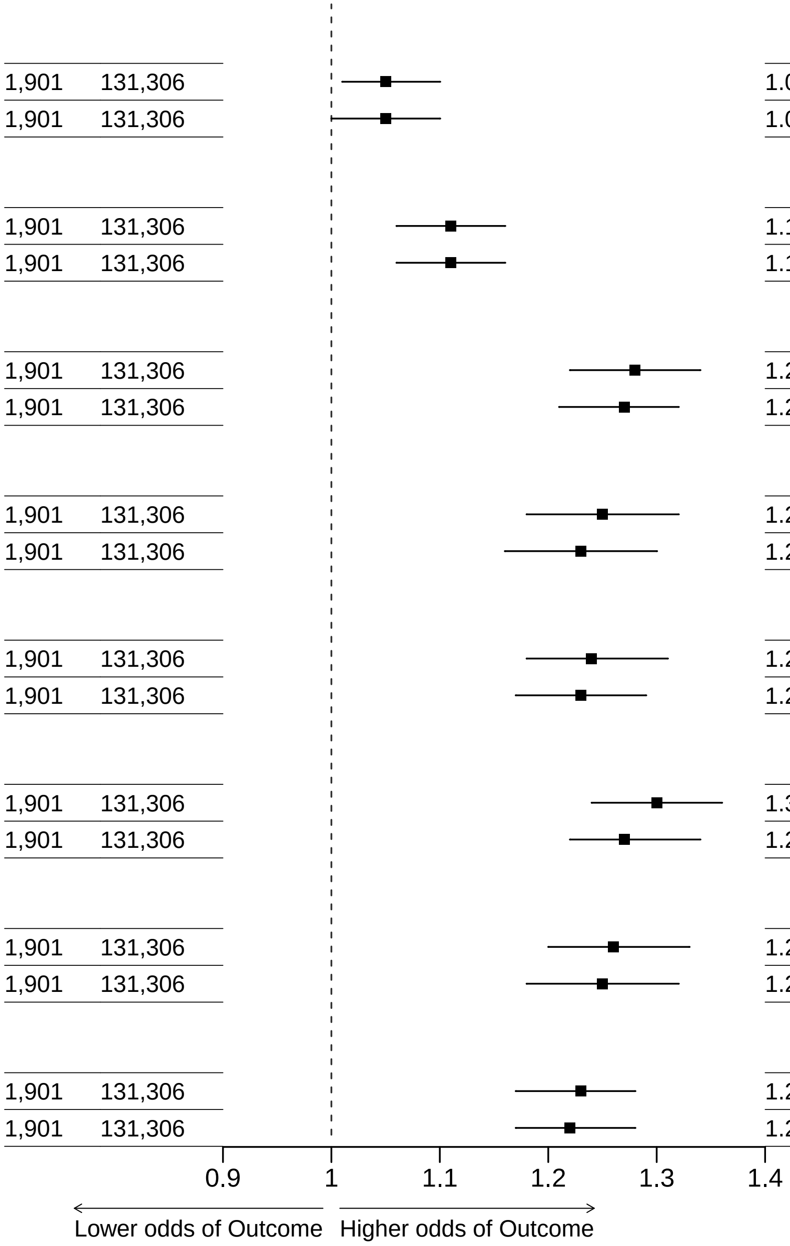

Webfigure 12: Odds of baseline self-reported angina or myocardial infarction per 1SD increase in each PRS, for participants aged 35-79 years at recruitment
Analyses as for Figure 2.

PRS name	Cases	Controls	OR per SD (95% CI)	AUC (95%CI)
**Tada et al/PGS000011/ European/44 SNPs**				
Partially adjusted	4,649	128,558	1.05 (1.02−1.08)	0.718 (0.711−0.724)
Fully adjusted	4,649	128,558	1.04 (1.01−1.07)	0.750 (0.743−0.756)
**Oni−Orisan et al/PGS004595/ European/141 SNPs**				
Partially adjusted	4,649	128,558	1.10 (1.06−1.13)	0.718 (0.711−0.725)
Fully adjusted	4,649	128,558	1.09 (1.06−1.12)	0.750 (0.743−0.757)
**Koyama et al/PGS000337/ Japanese and European/64,185 SNPs**				
Partially adjusted	4,649	128,558	1.25 (1.21−1.28)	0.723 (0.716−0.729)
Fully adjusted	4,649	128,558	1.21 (1.18−1.25)	0.753 (0.746−0.759)
**Tcheandjieu et al/PGS003446/ Multi−ancestry/485,464 SNPs**				
Partially adjusted	4,649	128,558	1.21 (1.17−1.26)	0.720 (0.714−0.727)
Fully adjusted	4,649	128,558	1.18 (1.13−1.22)	0.751 (0.745−0.758)
**Tamlander et al/ PGS001780/ European/1,087,958 SNPs**				
Partially adjusted	4,649	128,558	1.23 (1.19−1.27)	0.722 (0.715−0.728)
Fully adjusted	4,649	128,558	1.20 (1.17−1.24)	0.752 (0.745−0.759)
**Patel et al/PGS003725/ Multi−ancestry/1,273,824 SNPs**				
Partially adjusted	4,649	128,558	1.30 (1.26−1.34)	0.725 (0.718−0.732)
Fully adjusted	4,649	128,558	1.25 (1.21−1.28)	0.753 (0.747−0.760)
**Inouye et al/PGS000018/ European/1,720,068 SNPs**				
Partially adjusted	4,649	128,558	1.24 (1.19−1.28)	0.721 (0.714−0.728)
Fully adjusted	4,649	128,558	1.21 (1.17−1.25)	0.752 (0.745−0.758)
**Khera et al/PGS000013/ European/6,472,620 SNPs**				
Partially adjusted	4,649	128,558	1.20 (1.16−1.23)	0.721 (0.714−0.728)
Fully adjusted	4,649	128,558	1.18 (1.15−1.22)	0.752 (0.745−0.758)

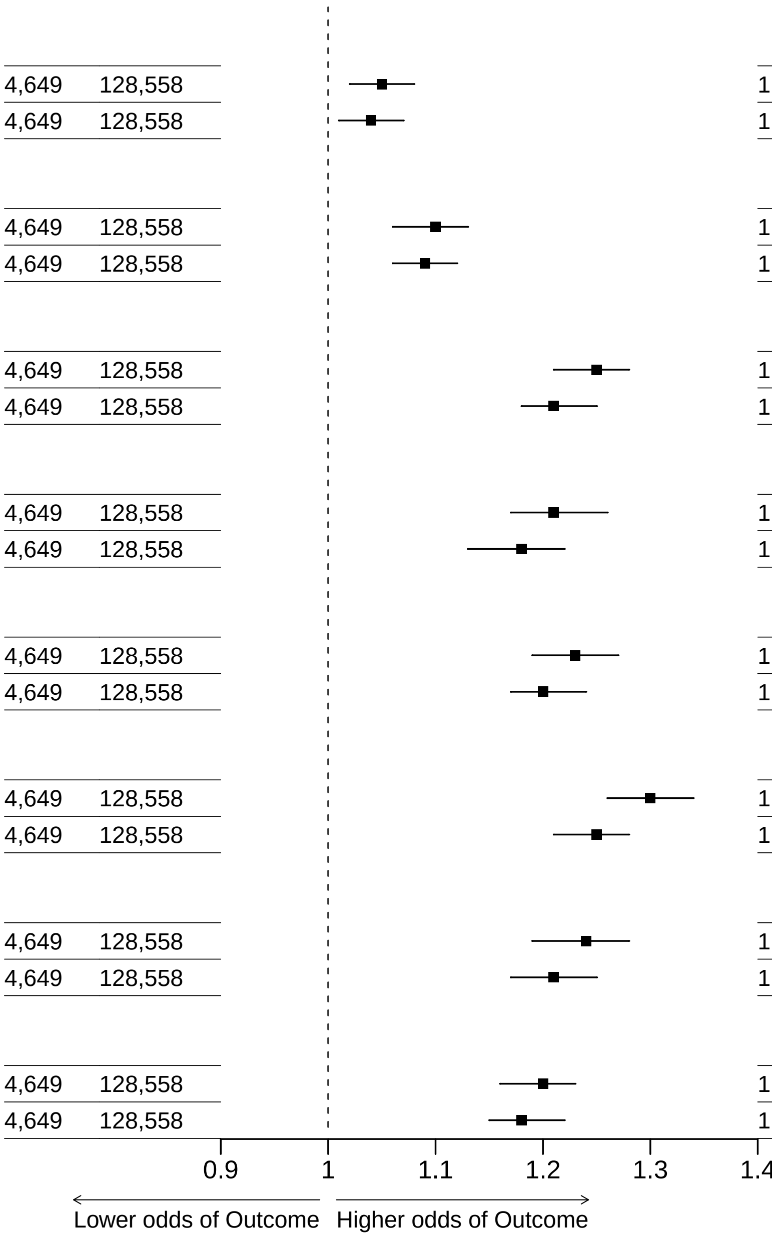

Webfigure 13: Odds of CHD (now defined as baseline self-reported angina or myocardial infarction, or death before age 80 with CHD listed as the primary cause of death) per 1SD increase in each PRS, for participants aged 35-79 years at recruitment
Analyses as for Figure 2.

PRS name	Cases	Controls	OR per SD (95% CI)	AUC (95%CI)
**Tada et al/PGS000011/ European/44 SNPs**				
Partially adjusted	3,479	129,728	1.06 (1.02−1.09)	0.705 (0.697−0.713)
Fully adjusted	3,479	129,728	1.05 (1.01−1.08)	0.748 (0.741−0.756)
**Oni−Orisan et al/PGS004595/ European/141 SNPs**				
Partially adjusted	3,479	129,728	1.10 (1.06−1.14)	0.706 (0.698−0.713)
Fully adjusted	3,479	129,728	1.09 (1.06−1.13)	0.748 (0.741−0.756)
**Koyama et al/PGS000337/ Japanese and European/64,185 SNPs**				
Partially adjusted	3,479	129,728	1.21 (1.17−1.25)	0.709 (0.702−0.717)
Fully adjusted	3,479	129,728	1.17 (1.13−1.21)	0.750 (0.742−0.757)
**Tcheandjieu et al/PGS003446/ Multi−ancestry/485,464 SNPs**				
Partially adjusted	3,479	129,728	1.17 (1.13−1.22)	0.707 (0.700−0.715)
Fully adjusted	3,479	129,728	1.13 (1.09−1.18)	0.749 (0.741−0.756)
**Tamlander et al/ PGS001780/ European/1,087,958 SNPs**				
Partially adjusted	3,479	129,728	1.22 (1.17−1.26)	0.709 (0.701−0.717)
Fully adjusted	3,479	129,728	1.18 (1.14−1.23)	0.750 (0.743−0.757)
**Patel et al/PGS003725/ Multi−ancestry/1,273,824 SNPs**				
Partially adjusted	3,479	129,728	1.30 (1.25−1.34)	0.714 (0.706−0.721)
Fully adjusted	3,479	129,728	1.23 (1.19−1.28)	0.752 (0.744−0.759)
**Inouye et al/PGS000018/ European/1,720,068 SNPs**				
Partially adjusted	3,479	129,728	1.21 (1.16−1.26)	0.708 (0.700−0.715)
Fully adjusted	3,479	129,728	1.17 (1.13−1.22)	0.749 (0.742−0.757)
**Khera et al/PGS000013/ European/6,472,620 SNPs**				
Partially adjusted	3,479	129,728	1.17 (1.13−1.21)	0.707 (0.700−0.715)
Fully adjusted	3,479	129,728	1.15 (1.11−1.19)	0.749 (0.742−0.757)

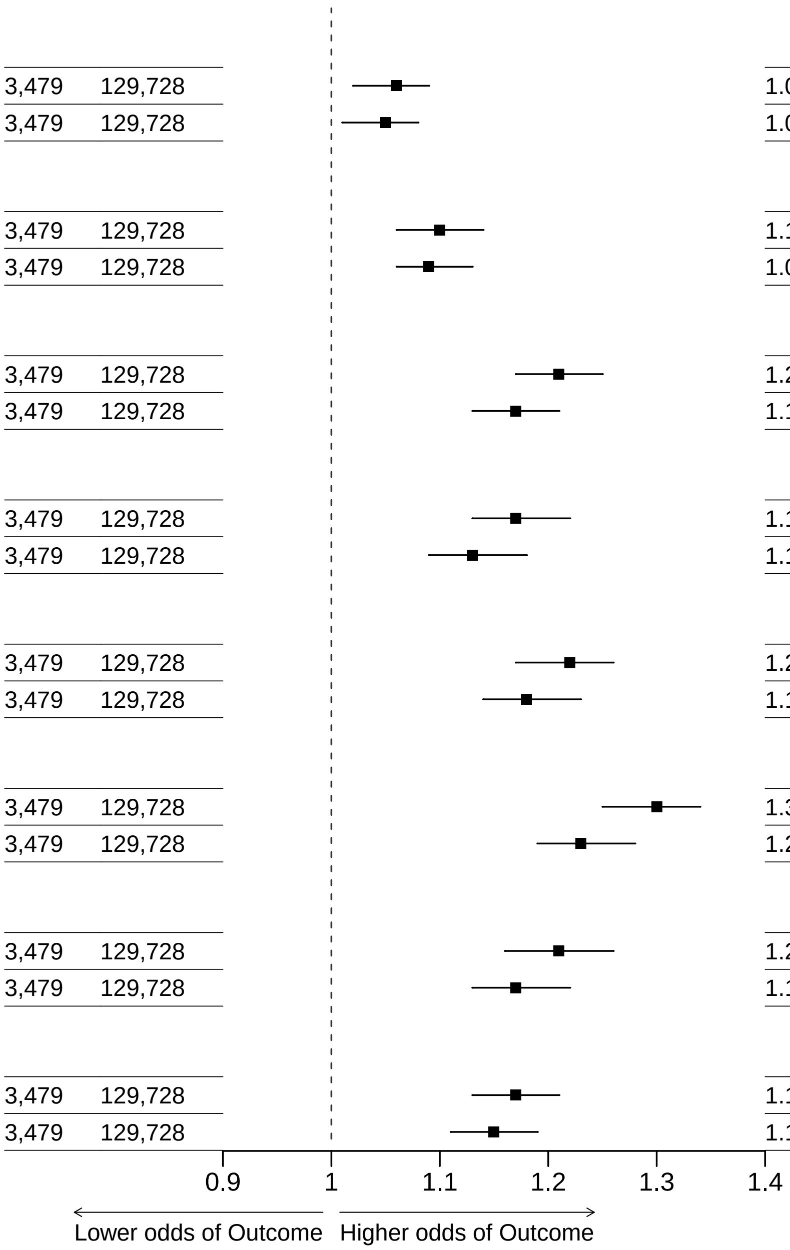

Webfigure 14: Odds of death before age 80 with CHD listed anywhere on the death certificate, per 1SD increase in each PRS
Analyses as for Figure 2.

PRS name	Cases	Controls	OR per SD (95% CI)	AUC (95%CI)
**Tada et al/PGS000011/ European/44 SNPs**				
Partially adjusted	2,927	130,280	1.05 (1.01−1.09)	0.704 (0.696−0.712)
Fully adjusted	2,927	130,280	1.04 (1.00−1.08)	0.756 (0.748−0.764)
**Oni−Orisan et al/PGS004595/ European/141 SNPs**				
Partially adjusted	2,927	130,280	1.09 (1.05−1.13)	0.704 (0.696−0.712)
Fully adjusted	2,927	130,280	1.08 (1.04−1.12)	0.756 (0.748−0.764)
**Koyama et al/PGS000337/ Japanese and European/64,185 SNPs**				
Partially adjusted	2,927	130,280	1.23 (1.19−1.28)	0.709 (0.701−0.717)
Fully adjusted	2,927	130,280	1.19 (1.14−1.23)	0.758 (0.750−0.766)
**Tcheandjieu et al/PGS003446/ Multi−ancestry/485,464 SNPs**				
Partially adjusted	2,927	130,280	1.20 (1.14−1.25)	0.707 (0.698−0.715)
Fully adjusted	2,927	130,280	1.15 (1.10−1.20)	0.756 (0.748−0.764)
**Tamlander et al/ PGS001780/ European/1,087,958 SNPs**				
Partially adjusted	2,927	130,280	1.22 (1.18−1.27)	0.708 (0.700−0.716)
Fully adjusted	2,927	130,280	1.19 (1.14−1.24)	0.757 (0.749−0.765)
**Patel et al/PGS003725/ Multi−ancestry/1,273,824 SNPs**				
Partially adjusted	2,927	130,280	1.31 (1.26−1.36)	0.713 (0.705−0.722)
Fully adjusted	2,927	130,280	1.23 (1.19−1.28)	0.759 (0.751−0.767)
**Inouye et al/PGS000018/ European/1,720,068 SNPs**				
Partially adjusted	2,927	130,280	1.23 (1.18−1.29)	0.707 (0.699−0.715)
Fully adjusted	2,927	130,280	1.19 (1.14−1.24)	0.757 (0.749−0.765)
**Khera et al/PGS000013/ European/6,472,620 SNPs**				
Partially adjusted	2,927	130,280	1.18 (1.14−1.23)	0.707 (0.699−0.715)
Fully adjusted	2,927	130,280	1.16 (1.12−1.21)	0.757 (0.749−0.765)

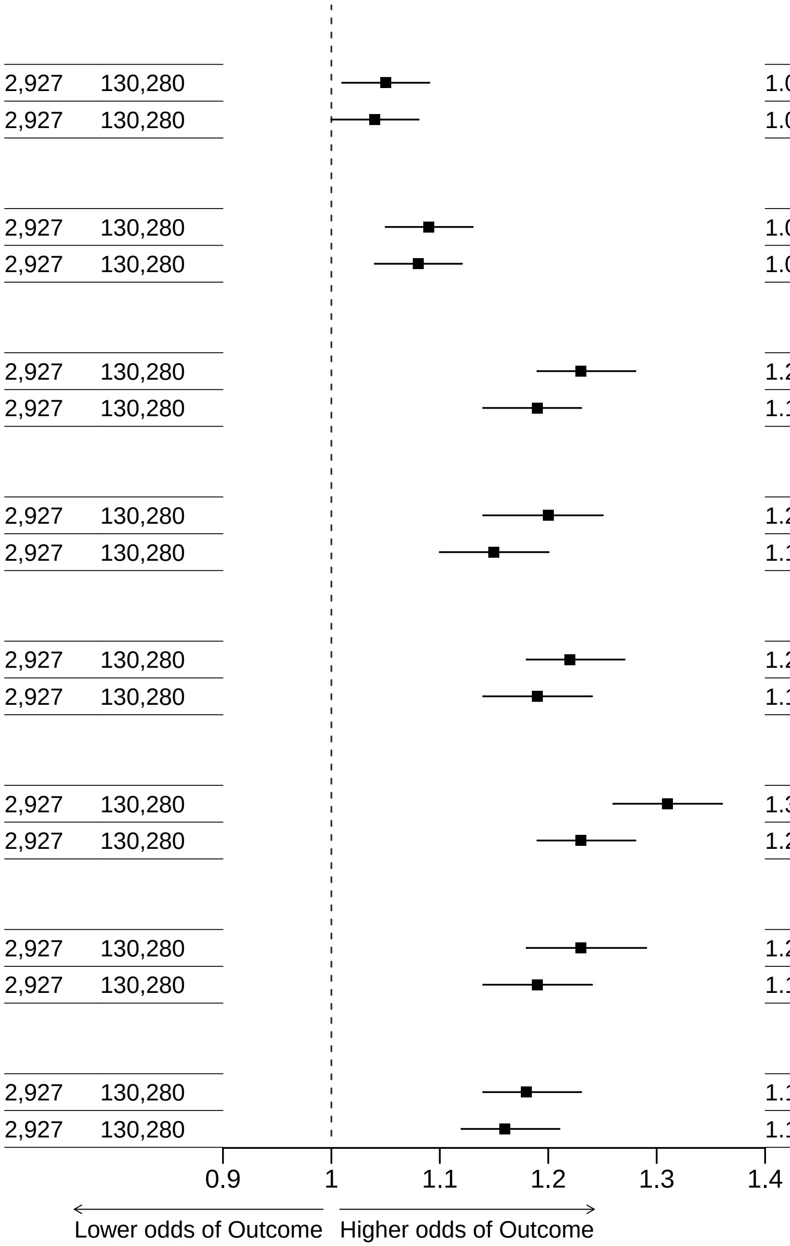

Webfigure 15: Odds of death before age 80 with CHD listed as the primary cause on the death certificate, per 1SD increase in each PRS
Analyses as for Figure 2.

Analysis outcome	Cases	Controls	OR per SD (95% CI)	AUC (95%CI)
**Tada et al/PGS000011/ European/44 SNPs**				
Primary definition of CHD	5,163	128,044	1.05 (1.03−1.08)	0.716 (0.710−0.723)
Baseline angina	494	132,713	1.07 (0.98−1.17)	0.741 (0.720−0.762)
Baseline myocardial infarction	1,476	131,731	1.05 (1.00−1.11)	0.758 (0.745−0.770)
Baseline CHD	1,901	131,306	1.05 (1.01−1.10)	0.748 (0.738−0.759)
Baseline CHD or CHD death (primary cause)	4,649	128,558	1.05 (1.02−1.08)	0.718 (0.711−0.724)
CHD death (anywhere on the death certificate)	3,479	129,728	1.06 (1.02−1.09)	0.705 (0.697−0.713)
CHD death (primary cause on death certificate)	2,927	130,280	1.05 (1.01−1.09)	0.704 (0.696−0.712)
Primary definition (among unrelated participants)	2,947	72,928	1.06 (1.02−1.10)	0.715 (0.707−0.724)
**Oni−Orisan et al/PGS004595/ European/141 SNPs**				
Primary definition of CHD	5,163	128,044	1.10 (1.07−1.13)	0.717 (0.711−0.723)
Baseline angina	494	132,713	1.05 (0.96−1.15)	0.740 (0.719−0.761)
Baseline myocardial infarction	1,476	131,731	1.13 (1.08−1.19)	0.759 (0.746−0.771)
Baseline CHD	1,901	131,306	1.11 (1.06−1.16)	0.749 (0.738−0.760)
Baseline CHD or CHD death (primary cause)	4,649	128,558	1.10 (1.06−1.13)	0.718 (0.711−0.725)
CHD death (anywhere on the death certificate)	3,479	129,728	1.10 (1.06−1.14)	0.706 (0.698−0.713)
CHD death (primary cause on death certificate)	2,927	130,280	1.09 (1.05−1.13)	0.704 (0.696−0.712)
Primary definition (among unrelated participants)	2,947	72,928	1.09 (1.05−1.13)	0.716 (0.707−0.724)
**Koyama et al/PGS000337/ Japanese and European/64,185 SNPs**				
Primary definition of CHD	5,163	128,044	1.23 (1.19−1.26)	0.721 (0.714−0.727)
Baseline angina	494	132,713	1.21 (1.11−1.33)	0.744 (0.722−0.765)
Baseline myocardial infarction	1,476	131,731	1.30 (1.24−1.37)	0.764 (0.751−0.776)
Baseline CHD	1,901	131,306	1.28 (1.22−1.34)	0.754 (0.743−0.765)
Baseline CHD or CHD death (primary cause)	4,649	128,558	1.25 (1.21−1.28)	0.723 (0.716−0.729)
CHD death (anywhere on the death certificate)	3,479	129,728	1.21 (1.17−1.25)	0.709 (0.702−0.717)
CHD death (primary cause on death certificate)	2,927	130,280	1.23 (1.19−1.28)	0.709 (0.701−0.717)
Primary definition (among unrelated participants)	2,947	72,928	1.23 (1.19−1.28)	0.720 (0.712−0.729)
**Tcheandjieu et al/PGS003446/ Multi−ancestry/485,464 SNPs**				
Primary definition of CHD	5,163	128,044	1.19 (1.15−1.23)	0.719 (0.712−0.725)
Baseline angina	494	132,713	1.11 (1.00−1.24)	0.741 (0.719−0.762)
Baseline myocardial infarction	1,476	131,731	1.30 (1.22−1.38)	0.762 (0.750−0.775)
Baseline CHD	1,901	131,306	1.25 (1.18−1.32)	0.751 (0.741−0.762)
Baseline CHD or CHD death (primary cause)	4,649	128,558	1.21 (1.17−1.26)	0.720 (0.714−0.727)
CHD death (anywhere on the death certificate)	3,479	129,728	1.17 (1.13−1.22)	0.707 (0.700−0.715)
CHD death (primary cause on death certificate)	2,927	130,280	1.20 (1.14−1.25)	0.707 (0.698−0.715)
Primary definition (among unrelated participants)	2,947	72,928	1.20 (1.14−1.25)	0.718 (0.710−0.727)
**Tamlander et al/ PGS001780/ European/1,087,958 SNPs**				
Primary definition of CHD	5,163	128,044	1.22 (1.19−1.26)	0.720 (0.714−0.727)
Baseline angina	494	132,713	1.11 (1.01−1.22)	0.741 (0.720−0.762)
Baseline myocardial infarction	1,476	131,731	1.30 (1.23−1.37)	0.762 (0.750−0.775)
Baseline CHD	1,901	131,306	1.24 (1.18−1.31)	0.752 (0.741−0.763)
Baseline CHD or CHD death (primary cause)	4,649	128,558	1.23 (1.19−1.27)	0.722 (0.715−0.728)
CHD death (anywhere on the death certificate)	3,479	129,728	1.22 (1.17−1.26)	0.709 (0.701−0.717)
CHD death (primary cause on death certificate)	2,927	130,280	1.22 (1.18−1.27)	0.708 (0.700−0.716)
Primary definition (among unrelated participants)	2,947	72,928	1.22 (1.17−1.27)	0.719 (0.710−0.728)
**Patel et al/PGS003725/ Multi−ancestry/1,273,824 SNPs**				
Primary definition of CHD	5,163	128,044	1.29 (1.25−1.33)	0.724 (0.717−0.730)
Baseline angina	494	132,713	1.15 (1.05−1.25)	0.742 (0.721−0.763)
Baseline myocardial infarction	1,476	131,731	1.36 (1.30−1.44)	0.765 (0.752−0.778)
Baseline CHD	1,901	131,306	1.30 (1.24−1.36)	0.753 (0.742−0.765)
Baseline CHD or CHD death (primary cause)	4,649	128,558	1.30 (1.26−1.34)	0.725 (0.718−0.732)
CHD death (anywhere on the death certificate)	3,479	129,728	1.30 (1.25−1.34)	0.714 (0.706−0.721)
CHD death (primary cause on death certificate)	2,927	130,280	1.31 (1.26−1.36)	0.713 (0.705−0.722)
Primary definition (among unrelated participants)	2,947	72,928	1.29 (1.25−1.34)	0.723 (0.714−0.731)
**Inouye et al/PGS000018/ European/1,720,068 SNPs**				
Primary definition of CHD	5,163	128,044	1.22 (1.18−1.26)	0.719 (0.713−0.726)
Baseline angina	494	132,713	1.15 (1.04−1.28)	0.742 (0.721−0.763)
Baseline myocardial infarction	1,476	131,731	1.31 (1.23−1.39)	0.762 (0.749−0.774)
Baseline CHD	1,901	131,306	1.26 (1.20−1.33)	0.752 (0.741−0.763)
Baseline CHD or CHD death (primary cause)	4,649	128,558	1.24 (1.19−1.28)	0.721 (0.714−0.728)
CHD death (anywhere on the death certificate)	3,479	129,728	1.21 (1.16−1.26)	0.708 (0.700−0.715)
CHD death (primary cause on death certificate)	2,927	130,280	1.23 (1.18−1.29)	0.707 (0.699−0.715)
Primary definition (among unrelated participants)	2,947	72,928	1.23 (1.17−1.28)	0.718 (0.710−0.727)
**Khera et al/PGS000013/ European/6,472,620 SNPs**				
Primary definition of CHD	5,163	128,044	1.19 (1.15−1.22)	0.719 (0.713−0.726)
Baseline angina	494	132,713	1.16 (1.06−1.27)	0.744 (0.723−0.765)
Baseline myocardial infarction	1,476	131,731	1.26 (1.19−1.32)	0.762 (0.749−0.774)
Baseline CHD	1,901	131,306	1.23 (1.17−1.28)	0.752 (0.741−0.763)
Baseline CHD or CHD death (primary cause)	4,649	128,558	1.20 (1.16−1.23)	0.721 (0.714−0.728)
CHD death (anywhere on the death certificate)	3,479	129,728	1.17 (1.13−1.21)	0.707 (0.700−0.715)
CHD death (primary cause on death certificate)	2,927	130,280	1.18 (1.14−1.23)	0.707 (0.699−0.715)
Primary definition (among unrelated participants)	2,947	72,928	1.18 (1.14−1.22)	0.718 (0.709−0.726)

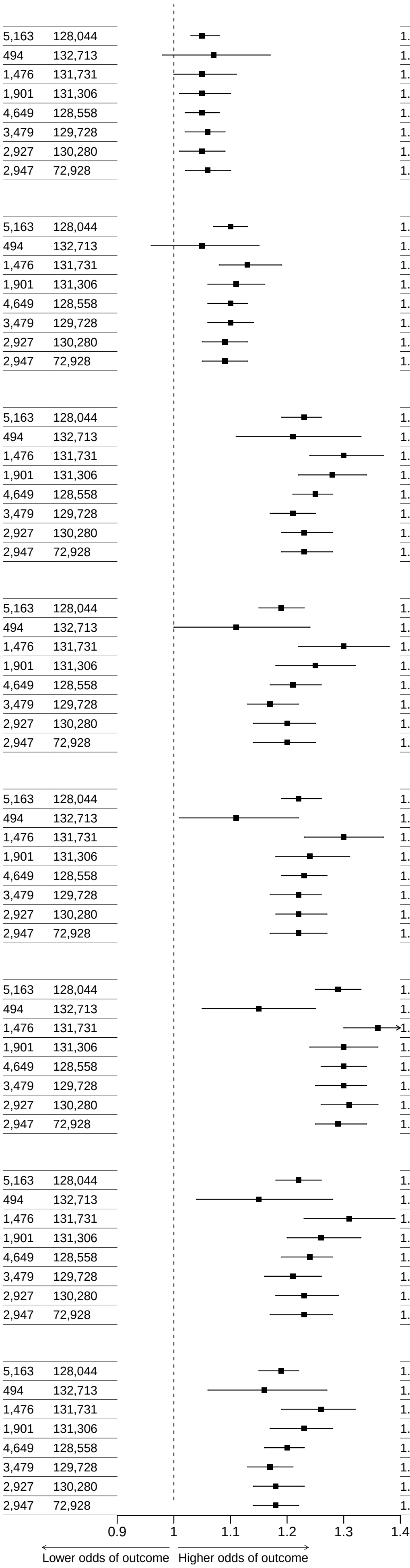

Webfigure 16: Sensitivity analyses (varying the CHD outcome) with partial adjustments
Odds ratios (ORs) were estimated with regression models adjusted for baseline age, sex and 7 genetic principal components.

Analysis outcome	Cases	Controls	OR per SD (95% CI)	AUC (95%CI)
**Tada et al/PGS000011/ European/44 SNPs**				
Primary definition of CHD	5,163	128,044	1.05 (1.02−1.08)	0.745 (0.739−0.752)
Baseline angina	494	132,713	1.07 (0.98−1.17)	0.748 (0.727−0.769)
Baseline myocardial infarction	1,476	131,731	1.05 (0.99−1.10)	0.771 (0.759−0.783)
Baseline CHD	1,901	131,306	1.05 (1.00−1.10)	0.760 (0.750−0.771)
Baseline CHD or CHD death (primary cause)	4,649	128,558	1.04 (1.01−1.07)	0.750 (0.743−0.756)
CHD death (anywhere on the death certificate)	3,479	129,728	1.05 (1.01−1.08)	0.748 (0.741−0.756)
CHD death (primary cause on death certificate)	2,927	130,280	1.04 (1.00−1.08)	0.756 (0.748−0.764)
Primary definition (among unrelated participants)	2,947	72,928	1.05 (1.01−1.09)	0.747 (0.739−0.755)
**Oni−Orisan et al/PGS004595/ European/141 SNPs**				
Primary definition of CHD	5,163	128,044	1.09 (1.06−1.13)	0.746 (0.739−0.752)
Baseline angina	494	132,713	1.05 (0.96−1.15)	0.747 (0.726−0.769)
Baseline myocardial infarction	1,476	131,731	1.13 (1.07−1.19)	0.771 (0.759−0.783)
Baseline CHD	1,901	131,306	1.11 (1.06−1.16)	0.761 (0.750−0.771)
Baseline CHD or CHD death (primary cause)	4,649	128,558	1.09 (1.06−1.12)	0.750 (0.743−0.757)
CHD death (anywhere on the death certificate)	3,479	129,728	1.09 (1.06−1.13)	0.748 (0.741−0.756)
CHD death (primary cause on death certificate)	2,927	130,280	1.08 (1.04−1.12)	0.756 (0.748−0.764)
Primary definition (among unrelated participants)	2,947	72,928	1.08 (1.04−1.12)	0.747 (0.738−0.755)
**Koyama et al/PGS000337/ Japanese and European/64,185 SNPs**				
Primary definition of CHD	5,163	128,044	1.20 (1.17−1.23)	0.748 (0.742−0.754)
Baseline angina	494	132,713	1.21 (1.10−1.32)	0.750 (0.729−0.772)
Baseline myocardial infarction	1,476	131,731	1.29 (1.22−1.36)	0.775 (0.763−0.788)
Baseline CHD	1,901	131,306	1.27 (1.21−1.32)	0.764 (0.753−0.775)
Baseline CHD or CHD death (primary cause)	4,649	128,558	1.21 (1.18−1.25)	0.753 (0.746−0.759)
CHD death (anywhere on the death certificate)	3,479	129,728	1.17 (1.13−1.21)	0.750 (0.742−0.757)
CHD death (primary cause on death certificate)	2,927	130,280	1.19 (1.14−1.23)	0.758 (0.750−0.766)
Primary definition (among unrelated participants)	2,947	72,928	1.21 (1.16−1.25)	0.750 (0.741−0.758)
**Tcheandjieu et al/PGS003446/ Multi−ancestry/485,464 SNPs**				
Primary definition of CHD	5,163	128,044	1.16 (1.12−1.20)	0.746 (0.740−0.753)
Baseline angina	494	132,713	1.11 (1.00−1.23)	0.748 (0.726−0.769)
Baseline myocardial infarction	1,476	131,731	1.28 (1.20−1.37)	0.774 (0.762−0.786)
Baseline CHD	1,901	131,306	1.23 (1.16−1.30)	0.763 (0.752−0.773)
Baseline CHD or CHD death (primary cause)	4,649	128,558	1.18 (1.13−1.22)	0.751 (0.745−0.758)
CHD death (anywhere on the death certificate)	3,479	129,728	1.13 (1.09−1.18)	0.749 (0.741−0.756)
CHD death (primary cause on death certificate)	2,927	130,280	1.15 (1.10−1.20)	0.756 (0.748−0.764)
Primary definition (among unrelated participants)	2,947	72,928	1.16 (1.11−1.22)	0.748 (0.740−0.757)
**Tamlander et al/ PGS001780/ European/1,087,958 SNPs**				
Primary definition of CHD	5,163	128,044	1.20 (1.16−1.23)	0.748 (0.741−0.754)
Baseline angina	494	132,713	1.10 (1.00−1.21)	0.748 (0.727−0.770)
Baseline myocardial infarction	1,476	131,731	1.29 (1.22−1.36)	0.774 (0.762−0.786)
Baseline CHD	1,901	131,306	1.23 (1.17−1.29)	0.763 (0.752−0.774)
Baseline CHD or CHD death (primary cause)	4,649	128,558	1.20 (1.17−1.24)	0.752 (0.745−0.759)
CHD death (anywhere on the death certificate)	3,479	129,728	1.18 (1.14−1.23)	0.750 (0.743−0.757)
CHD death (primary cause on death certificate)	2,927	130,280	1.19 (1.14−1.24)	0.757 (0.749−0.765)
Primary definition (among unrelated participants)	2,947	72,928	1.19 (1.15−1.24)	0.749 (0.740−0.757)
**Patel et al/PGS003725/ Multi−ancestry/1,273,824 SNPs**				
Primary definition of CHD	5,163	128,044	1.24 (1.20−1.28)	0.749 (0.743−0.755)
Baseline angina	494	132,713	1.14 (1.04−1.24)	0.749 (0.727−0.770)
Baseline myocardial infarction	1,476	131,731	1.34 (1.27−1.41)	0.776 (0.763−0.788)
Baseline CHD	1,901	131,306	1.27 (1.22−1.34)	0.763 (0.753−0.774)
Baseline CHD or CHD death (primary cause)	4,649	128,558	1.25 (1.21−1.28)	0.753 (0.747−0.760)
CHD death (anywhere on the death certificate)	3,479	129,728	1.23 (1.19−1.28)	0.752 (0.744−0.759)
CHD death (primary cause on death certificate)	2,927	130,280	1.23 (1.19−1.28)	0.759 (0.751−0.767)
Primary definition (among unrelated participants)	2,947	72,928	1.24 (1.20−1.29)	0.750 (0.741−0.758)
**Inouye et al/PGS000018/ European/1,720,068 SNPs**				
Primary definition of CHD	5,163	128,044	1.19 (1.15−1.23)	0.747 (0.741−0.753)
Baseline angina	494	132,713	1.14 (1.03−1.27)	0.749 (0.727−0.770)
Baseline myocardial infarction	1,476	131,731	1.30 (1.22−1.38)	0.774 (0.762−0.786)
Baseline CHD	1,901	131,306	1.25 (1.18−1.32)	0.763 (0.752−0.774)
Baseline CHD or CHD death (primary cause)	4,649	128,558	1.21 (1.17−1.25)	0.752 (0.745−0.758)
CHD death (anywhere on the death certificate)	3,479	129,728	1.17 (1.13−1.22)	0.749 (0.742−0.757)
CHD death (primary cause on death certificate)	2,927	130,280	1.19 (1.14−1.24)	0.757 (0.749−0.765)
Primary definition (among unrelated participants)	2,947	72,928	1.20 (1.14−1.25)	0.748 (0.740−0.757)
**Khera et al/PGS000013/ European/6,472,620 SNPs**				
Primary definition of CHD	5,163	128,044	1.17 (1.14−1.20)	0.747 (0.741−0.754)
Baseline angina	494	132,713	1.16 (1.06−1.26)	0.751 (0.729−0.772)
Baseline myocardial infarction	1,476	131,731	1.25 (1.19−1.31)	0.774 (0.762−0.786)
Baseline CHD	1,901	131,306	1.22 (1.17−1.28)	0.763 (0.752−0.774)
Baseline CHD or CHD death (primary cause)	4,649	128,558	1.18 (1.15−1.22)	0.752 (0.745−0.758)
CHD death (anywhere on the death certificate)	3,479	129,728	1.15 (1.11−1.19)	0.749 (0.742−0.757)
CHD death (primary cause on death certificate)	2,927	130,280	1.16 (1.12−1.21)	0.757 (0.749−0.765)
Primary definition (among unrelated participants)	2,947	72,928	1.17 (1.13−1.21)	0.748 (0.740−0.757)

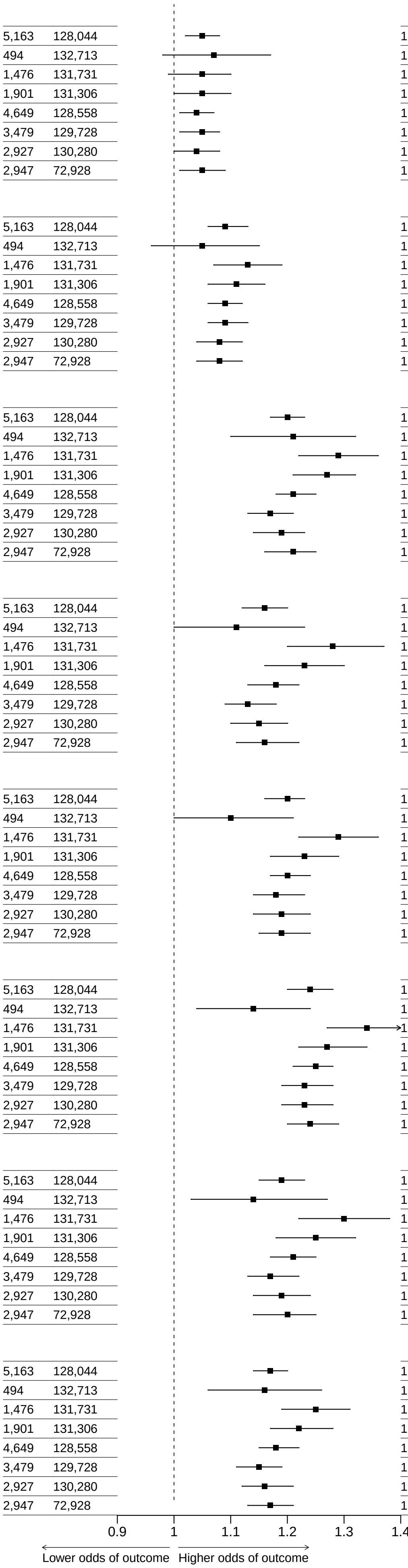

Webfigure 17: Sensitivity analyses (varying the CHD outcome) with full adjustments

Odds ratios (ORs) were estimated with regression models adjusted for baseline age, sex, 7 genetic principal components, waist-to-hip ratio, systolic and diastolic blood pressures, education attainment level, smoking status, and diabetes at baseline.

Patel et al/PGS003725/ Multi−ancestry/1,273,824 SNPs	Men			Women			Sex interaction p−value
	CHD case / control	OR per SD (95%CI)	AUC (95%CI)	CHD case / control	OR per SD (95%CI)	AUC (95%CI)	
**Partially adjusted**							
Primary definition of CHD	2,415 / 40,923	1.37 (1.32−1.43)	0.696 (0.686−0.706)	2,748 / 87,121	1.23 (1.18−1.28)	0.721 (0.712−0.730)	<0.0001
Baseline angina	136 / 43,202	1.43 (1.21−1.70)	0.718 (0.674−0.763)	358 / 89,511	1.05 (0.95−1.17)	0.756 (0.733−0.780)	4e−04
Baseline myocardial infarction	747 / 42,591	1.51 (1.40−1.62)	0.781 (0.765−0.796)	729 / 89,140	1.24 (1.15−1.33)	0.728 (0.709−0.747)	<0.0001
Baseline CHD	848 / 42,490	1.48 (1.38−1.59)	0.770 (0.755−0.786)	1,053 / 88,816	1.17 (1.10−1.25)	0.735 (0.720−0.750)	<0.0001
Baseline CHD or CHD death (primary cause)	2,165 / 41,173	1.39 (1.33−1.46)	0.698 (0.687−0.708)	2,484 / 87,385	1.23 (1.18−1.28)	0.724 (0.715−0.734)	<0.0001
CHD death (anywhere on the death certificate)	1,717 / 41,621	1.33 (1.27−1.40)	0.668 (0.656−0.679)	1,762 / 88,107	1.27 (1.21−1.33)	0.715 (0.704−0.725)	0.07
CHD death (primary cause on death certificate)	1,437 / 41,901	1.34 (1.27−1.42)	0.663 (0.650−0.675)	1,490 / 88,379	1.28 (1.21−1.34)	0.720 (0.709−0.731)	0.10
**Fully adjusted**							
Primary definition of CHD	2,415 / 40,923	1.33 (1.27−1.39)	0.729 (0.719−0.738)	2,748 / 87,121	1.17 (1.13−1.22)	0.747 (0.739−0.756)	<0.0001
Baseline angina	136 / 43,202	1.43 (1.21−1.70)	0.736 (0.693−0.779)	358 / 89,511	1.04 (0.94−1.16)	0.763 (0.739−0.787)	3e−04
Baseline myocardial infarction	747 / 42,591	1.49 (1.39−1.61)	0.792 (0.777−0.807)	729 / 89,140	1.21 (1.12−1.30)	0.744 (0.726−0.763)	<0.0001
Baseline CHD	848 / 42,490	1.47 (1.37−1.58)	0.781 (0.766−0.796)	1,053 / 88,816	1.14 (1.08−1.22)	0.748 (0.733−0.763)	<0.0001
Baseline CHD or CHD death (primary cause)	2,165 / 41,173	1.35 (1.29−1.41)	0.733 (0.723−0.744)	2,484 / 87,385	1.17 (1.12−1.22)	0.754 (0.745−0.763)	<0.0001
CHD death (anywhere on the death certificate)	1,717 / 41,621	1.27 (1.21−1.34)	0.717 (0.706−0.729)	1,762 / 88,107	1.19 (1.14−1.25)	0.754 (0.744−0.765)	0.01
CHD death (primary cause on death certificate)	1,437 / 41,901	1.28 (1.21−1.35)	0.722 (0.710−0.735)	1,490 / 88,379	1.20 (1.13−1.26)	0.767 (0.756−0.778)	0.02

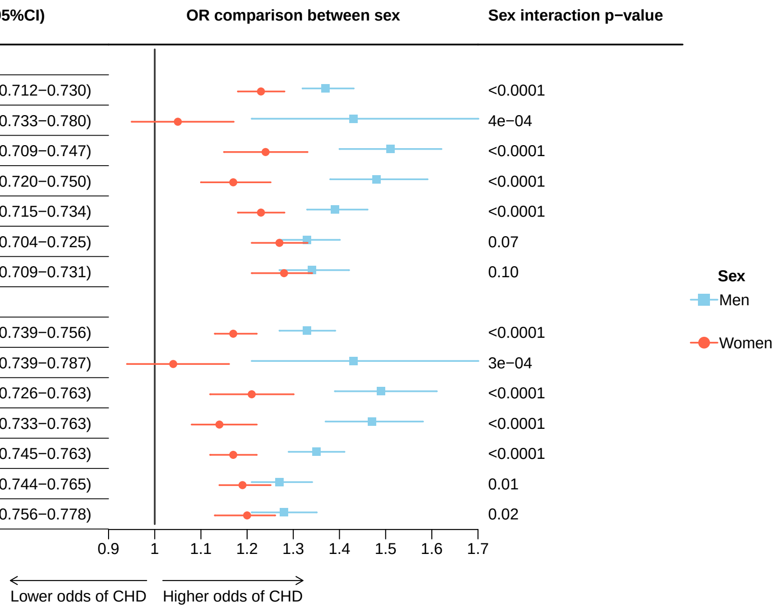

Webfigure 18: Odds of CHD (varying CHD outcomes) per 1SD increase for the Patel *et al.* PRS, by sex
Analyses as for Figure 2.

PRS name	CHD cases	CHD controls	OR per SD (95% CI)	AUC (95%CI)
**Tada et al/PGS000011/ European/44 SNPs**				
Partially adjusted	2,947	72,928	1.06 (1.02−1.10)	0.715 (0.707−0.724)
Fully adjusted	2,947	72,928	1.05 (1.01−1.09)	0.747 (0.739−0.755)
**Oni−Orisan et al/PGS004595/ European/141 SNPs**				
Partially adjusted	2,947	72,928	1.09 (1.05−1.13)	0.716 (0.707−0.724)
Fully adjusted	2,947	72,928	1.08 (1.04−1.12)	0.747 (0.738−0.755)
**Koyama et al/PGS000337/ Japanese and European/64,185 SNPs**				
Partially adjusted	2,947	72,928	1.23 (1.19−1.28)	0.720 (0.712−0.729)
Fully adjusted	2,947	72,928	1.21 (1.16−1.25)	0.750 (0.741−0.758)
**Tcheandjieu et al/PGS003446/ Multi−ancestry/485,464 SNPs**				
Partially adjusted	2,947	72,928	1.20 (1.14−1.25)	0.718 (0.710−0.727)
Fully adjusted	2,947	72,928	1.16 (1.11−1.22)	0.748 (0.740−0.757)
**Tamlander et al/ PGS001780/ European/1,087,958 SNPs**				
Partially adjusted	2,947	72,928	1.22 (1.17−1.27)	0.719 (0.710−0.728)
Fully adjusted	2,947	72,928	1.19 (1.15−1.24)	0.749 (0.740−0.757)
**Patel et al/PGS003725/ Multi−ancestry/1,273,824 SNPs**				
Partially adjusted	2,947	72,928	1.29 (1.25−1.34)	0.723 (0.714−0.731)
Fully adjusted	2,947	72,928	1.24 (1.20−1.29)	0.750 (0.741−0.758)
**Inouye et al/PGS000018/ European/1,720,068 SNPs**				
Partially adjusted	2,947	72,928	1.23 (1.17−1.28)	0.718 (0.710−0.727)
Fully adjusted	2,947	72,928	1.20 (1.14−1.25)	0.748 (0.740−0.757)
**Khera et al/PGS000013/ European/6,472,620 SNPs**				
Partially adjusted	2,947	72,928	1.18 (1.14−1.22)	0.718 (0.709−0.726)
Fully adjusted	2,947	72,928	1.17 (1.13−1.21)	0.748 (0.740−0.757)

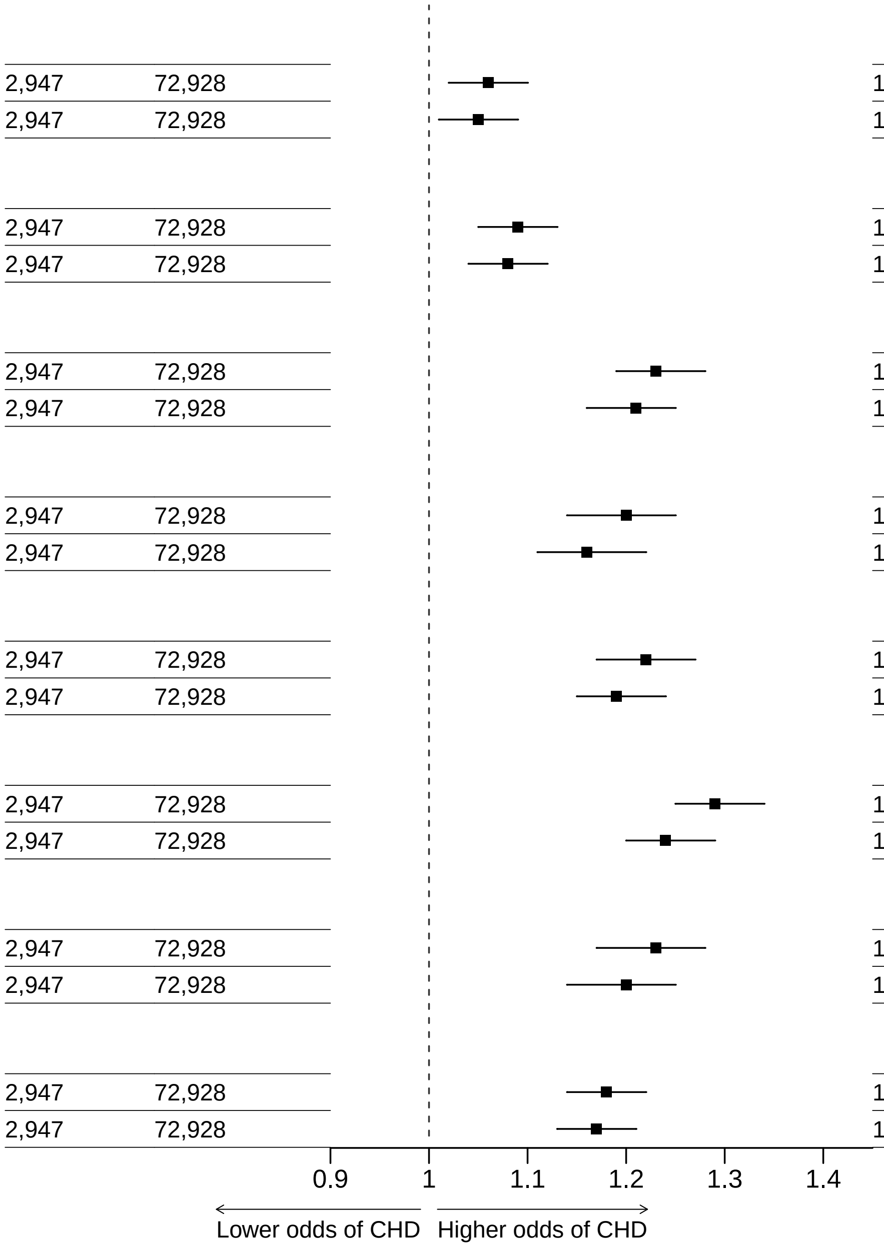

Webfigure 19: Sensitivity analyses (primary CHD definition) among participants unrelated to the 3rd degree
Analyses as for Figure 2.

PRS name	CHD cases	CHD controls	OR per SD (95% CI)	AUC (95%CI)
**Tada et al/PGS000011/ European/44 SNPs**				
Partially adjusted	7,155	130,236	1.06 (1.03−1.09)	0.762 (0.757−0.767)
Fully adjusted	7,155	130,236	1.06 (1.03−1.08)	0.778 (0.773−0.783)
**Oni−Orisan et al/PGS004595/ European/141 SNPs**				
Partially adjusted	7,155	130,236	1.09 (1.06−1.12)	0.763 (0.757−0.768)
Fully adjusted	7,155	130,236	1.08 (1.06−1.11)	0.778 (0.773−0.783)
**Koyama et al/PGS000337/ Japanese and European/64,185 SNPs**				
Partially adjusted	7,155	130,236	1.19 (1.16−1.22)	0.765 (0.760−0.770)
Fully adjusted	7,155	130,236	1.17 (1.14−1.20)	0.780 (0.775−0.785)
**Tcheandjieu et al/PGS003446/ Multi−ancestry/485,464 SNPs**				
Partially adjusted	7,155	130,236	1.15 (1.12−1.19)	0.764 (0.758−0.769)
Fully adjusted	7,155	130,236	1.13 (1.09−1.16)	0.779 (0.774−0.784)
**Tamlander et al/ PGS001780/ European/1,087,958 SNPs**				
Partially adjusted	7,155	130,236	1.19 (1.15−1.22)	0.764 (0.759−0.770)
Fully adjusted	7,155	130,236	1.16 (1.13−1.19)	0.779 (0.774−0.784)
**Patel et al/PGS003725/ Multi−ancestry/1,273,824 SNPs**				
Partially adjusted	7,155	130,236	1.24 (1.21−1.27)	0.766 (0.761−0.772)
Fully adjusted	7,155	130,236	1.20 (1.17−1.23)	0.780 (0.775−0.785)
**Inouye et al/PGS000018/ European/1,720,068 SNPs**				
Partially adjusted	7,155	130,236	1.19 (1.15−1.22)	0.764 (0.759−0.769)
Fully adjusted	7,155	130,236	1.16 (1.13−1.20)	0.779 (0.774−0.784)
**Khera et al/PGS000013/ European/6,472,620 SNPs**				
Partially adjusted	7,155	130,236	1.15 (1.12−1.18)	0.764 (0.759−0.769)
Fully adjusted	7,155	130,236	1.14 (1.11−1.17)	0.779 (0.774−0.784)

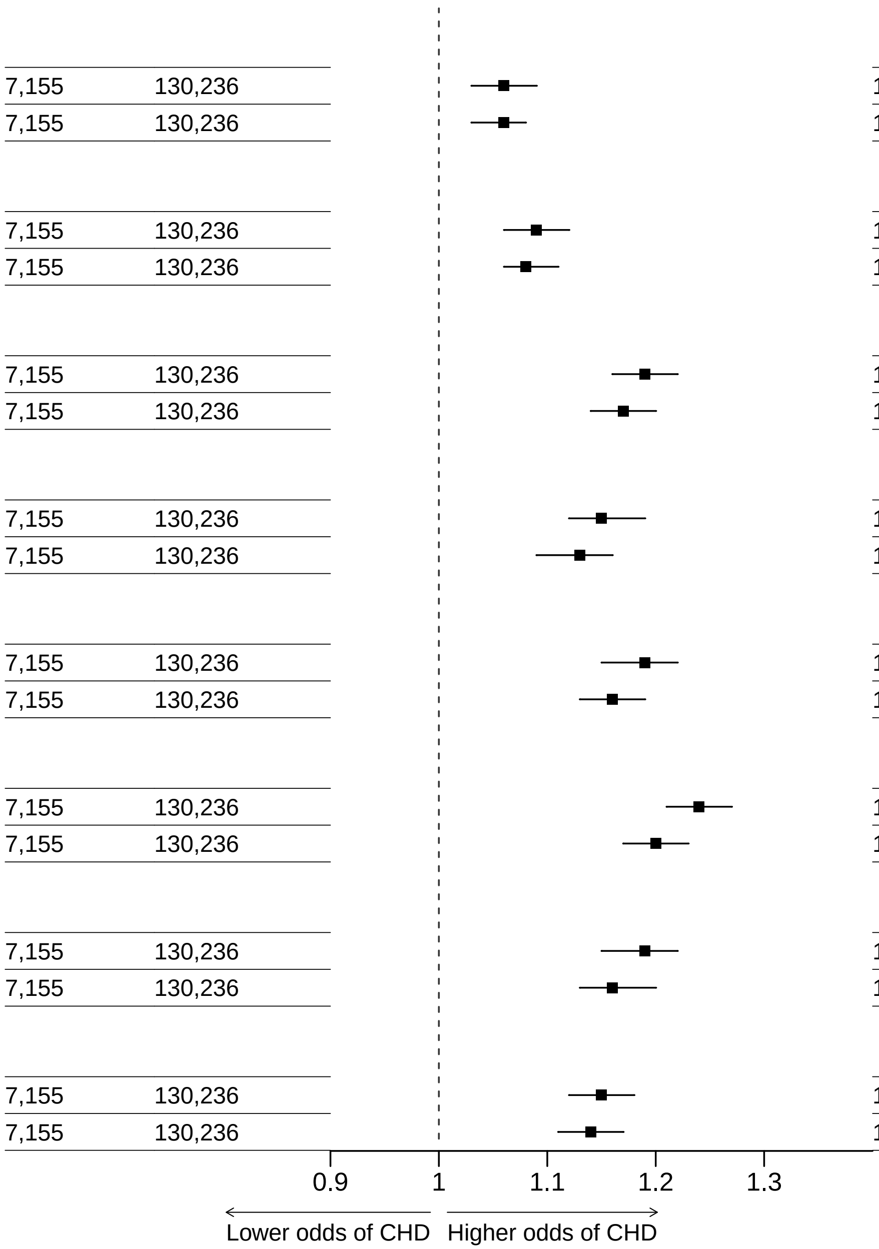

Webfigure 20: Odds of CHD before age 90 years per 1SD increase in each PRS, among participants age 35-89 at recruitment
Analyses as for Figure 2.

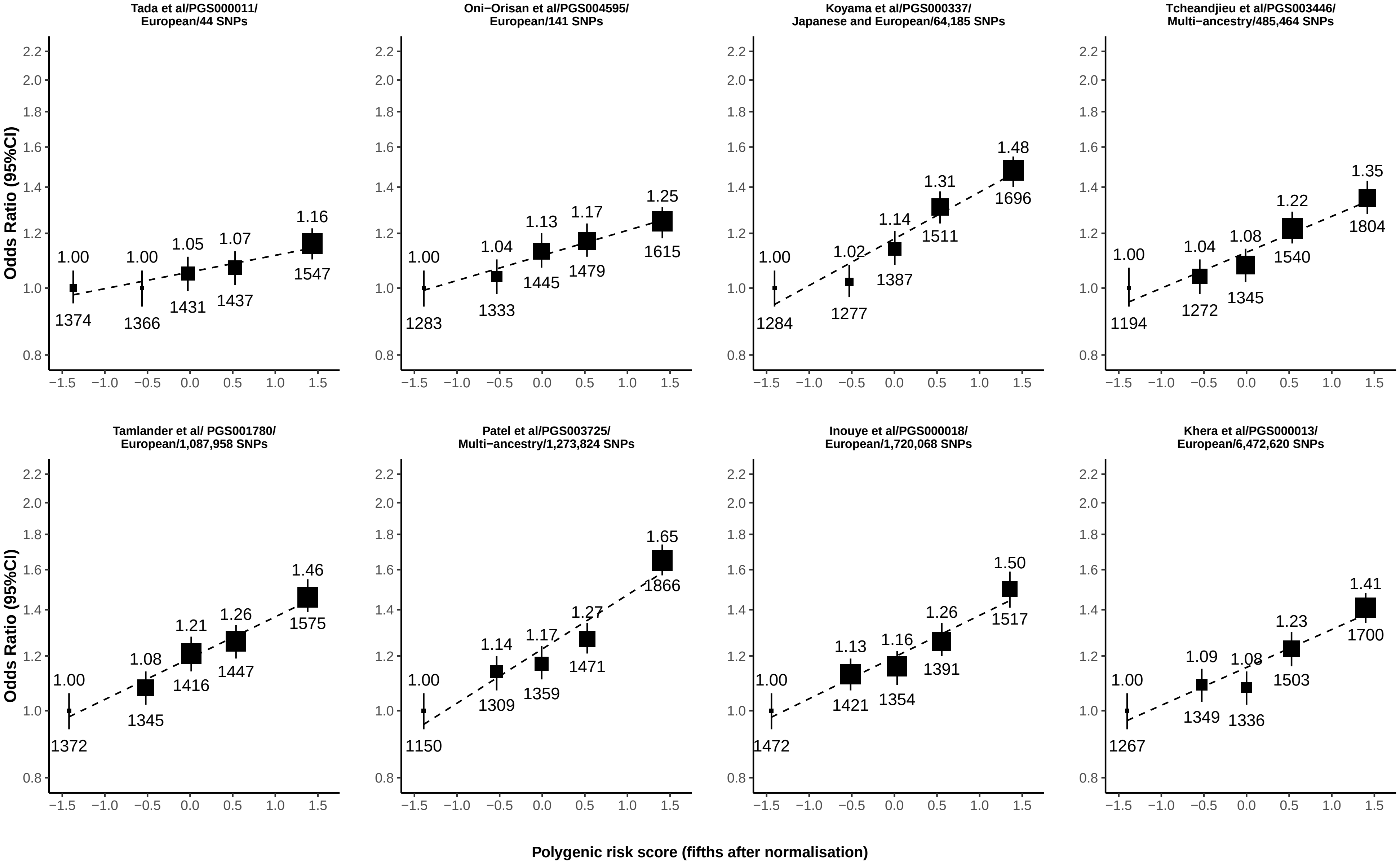

Webfigure 21: Odds of CHD before age 90 years by fifth of each PRS, among participants age 35-89 years at recruitment
Analyses as for Figure 1.
